# Bile acid supplementation in a high-fat diet improved growth performance, lipid deposition, ferroptosis, and intestinal health in Pacific white shrimp (*Litopenaeus vannamei*)

**DOI:** 10.1016/j.aninu.2025.09.020

**Published:** 2026-05-07

**Authors:** Kangyuan Qu, Tengfei Zhao, Yufei Chen, Yunzhen Li, Beiping Tan, Shiwei Xie

**Affiliations:** a Laboratory of Aquatic Animal Nutrition and Feed, Fisheries College, Guangdong Ocean University, Zhanjiang 524000, Guangdong, China; b Key Laboratory of Aquatic Feed Science and Technology for Livestock and Poultry in Southern China, Under the Ministry of Agriculture, Zhanjiang 524000, Guangdong, China; c Key Laboratory of Control for Disease of Aquatic Animals of Guangdong Higher Education Institutes, Zhanjiang 524000, Guangdong, China

**Keywords:** Bile acid, High-fat diet, Ferroptosis, Intestinal health, Pacific white shrimp

## Abstract

Emerging evidence highlights bile acid (BA) as a key regulator of lipid homeostasis in shrimp, enhancing growth performance in aquaculture. This study aimed to investigate the effects of dietary BA supplementation in high-fat diets on growth performance, lipid deposition, intestinal health, and ferroptosis in *Litopenaeus vannamei*. An 8-week feeding trial was conducted using, eight different diets formulated with two lipid levels (7% and 11%) and four BA supplementation levels (0, 200, 400, and 800 mg/kg). The diets were named as LFD, LF200, LF400, LF800, HFD, HF200, HF400, and HF800, respectively. A total of 1280 shrimp (0.52 ± 0.01 g) were divided into eight groups with four replicates, stocking 40 shrimp in each replicate tank. Shrimp fed high-fat diets exhibited comparable final body weight (FBW) and weight gain rate (WGR) compared to those fed low-fat diets. However, shrimp receiving 400 mg/kg BA-supplemented diets demonstrated a significant increase in FBW and WGR compared to the high-fat control group (*P* < 0.05). Compared to low-fat diets, high-fat diets significantly elevated whole-body lipid content in shrimp and increased hepatopancreatic levels of total cholesterol (T-CHO), triglycerides (TG), and nonesterified fatty acids (NEFA) (*P* < 0.05). Additionally, feeding high-fat diets upregulated genes associated with lipid metabolism, such as *ampk*, *fabp*, and *srebp*, in both hepatopancreas and intestines (*P* < 0.05). However, the addition of BA was effective in reducing the above indicators (*P* < 0.05). Transmission electron microscopy revealed that high-fat diets led to mitochondrial abnormalities in the intestine, including the disappearance of mitochondrial cristae. Immunostaining of the intestine confirmed that high-fat diet markedly reduce the protein expression of GPX4, and the addition of BA significantly up-regulated ferroptosis-related genes—*gpx4* and *fettrin*, enhanced GPX4 protein expression, downregulated the gene expression of *acsl4*, and reduced the malondialdehyde (MDA) content (*P* < 0.05). Moreover, the addition of BA to high-fat diets restructured the intestinal microbiota, improved intestinal histological structure, and increased the diversity indices of the intestinal microbiota. It also elevated the levels of *Tenacibaculum*, *Motilimona*s, *Ruegeria*, and significantly reduced the relative abundance of *Kurthia* (*P* < 0.05). These findings indicated that dietary BA supplementation may enhance growth performance, lipid metabolism, ferroptosis, and intestinal health of shrimp while mitigating the negative effects of a high-fat diet.

## 1 Introduction

The Pacific white shrimp (*Litopenaeus vannamei*), the most extensively cultivated crustacean globally, accounts for 80% of global shrimp production. As a major commodity in international aquaculture trade, its production reached 5.17 million tons in 2022 (Chen et al., 2024; Huang et al., 2024). To mitigate the high costs of fishmeal-based feeds, the industry has increasingly incorporated dietary lipids as an efficient protein-sparing strategy (Basto-Silva et al., 2022). However, optimal dietary lipid levels are highly stage-specific. For instance, postlarvae shrimp (1.7 mg) exhibit optimal survival and antioxidant capacity at 11.8% dietary lipid (Xie et al., 2019), while juvenile shrimp (3.0 g) achieve maximal growth when fed 12% lipid for 30 d or 10% lipid for 60 d (Zhang et al., 2013). For early juveniles (0.95–2.00 g), studies consistently report superior growth at 6%–9% lipid across different culture systems (González-Félix et al., 2003; Xu et al., 2018), whereas levels ≥ 12% may induce oxidative stress. Although high-fat feeds reduce costs (Yin et al., 2021), they can also promote hepatopancreatic lipid accumulation, leading to metabolic dysregulation (Chen et al., 2022). Given these trade-offs, dietary lipid levels of 7% and 11% were selected to evaluate the potential of bile acid (BA) supplementation to ameliorate these adverse effects.

Bile acids are endogenous sterol-derived molecules that play essential roles in lipid emulsification and absorption in vertebrates (Xu et al., 2022b). Interestingly, crustaceans lack the capacity to synthesize BA endogenously, making dietary supplementation particularly relevant (Su et al., 2022). Their amphiphilic nature enables efficient fat digestion through micelle formation (Faustino et al., 2016), and BA supplementation has documented benefits for shrimp growth performance and lipid metabolism (Wang et al., 2023). However, the interactive effects of BA supplementation across varying dietary lipid levels remain uncharacterized in *L. vannamei.*

The intestinal barrier serves as the primary defense against pathogens in shrimp, and its integrity hinges on structural robustness, immune competence, and microbial homeostasis (Duan et al., 2019). The BA exert multifaceted effects on this system: that directly enhance intestinal morphology and digestive efficiency (Su et al., 2021), while concurrently modulating microbial composition. Notably, BA supplementation increases the abundance of beneficial microbiota (Zhao et al., 2025) and counteracts dysbiosis induced by plant-based proteins (Li et al., 2023b). This regulatory function stems partly from bacterial metabolism of BA into bioactive compounds that optimize both lipid utilization and microbial homeostasis (Shi et al., 2022a).

Emerging evidence suggests that gut dysbiosis may predispose hosts to ferroptosis by exacerbating oxidative stress, a mechanism particularly relevant under high-fat diet conditions (Xue et al., 2024). Although ferroptosis remains understudied in penaeid shrimp, high-fat diets promote reactive oxygen species (ROS) accumulation and hepatopancreatic damage in *L. vannamei* (Zhang et al., 2013), consistent with ferroptosis-like pathways observed in mammals (Le et al., 2021). High-fat diets disrupt mitochondrial bioenergetics, the primary source of ROS in colonic epithelial cells, which aligns with ferroptosis, a cell death pathway mediated by ROS-driven phospholipid peroxidation that causes membrane integrity loss (Schriever et al., 2017; Yoo et al., 2021). Nevertheless, research on the effects of high dietary lipid on intestinal health and ferroptosis in crustaceans is still in its infancy compared to aquatic vertebrates (Xia et al., 2021; Yokota et al., 2012).

This study aimed to investigate the effects of BA supplementation and dietary lipid levels on shrimp growth performance, lipid metabolism, ferroptosis, and intestinal microbiota dynamics. A particular focus was placed on elucidating the potential of BA to mitigate high-fat diet-induced metabolic dysregulation through modulation of lipid metabolic pathways.

## 2 Material and methods

### 2.1 Animal ethics statement

The acquisition of experimental shrimp was authorized by Guangdong Ocean University's Animal Ethics Review Committee (permission number: GOU-IACUC-20240-A0326), and all studies were carried out in compliance with its regulations.

### 2.2 Experimental materials

Bile acid used in this study were purchased from Shandong Longchang Animal Health Product Co., Ltd. (Jinan, Shandong, China). The primary components of these BA were 68% porcine deoxycholic acid, 17% chenodeoxycholic acid, and 9% porcine cholic acid, with a total BA content of ≥ 95%. Their stability and efficacy have been validated in peer-reviewed studies evaluating the company's BA products (Qu et al., 2025; Yang et al., 2023b; Zhang et al., 2022b). This study formulated eight isonitrogenous diets, which included 7% lipid (LFD), 7% lipid + 200 mg/kg BA (LF200), 7% lipid + 400 mg/kg BA (LF400), 7% lipid + 800 mg/kg BA (LF800), 11% lipid (HFD), 11% lipid + 200 mg/kg BA (HF200), 11% lipid + 400 mg/kg BA (HF400), and 11% lipid + 800 mg/kg BA (HF800). The primary sources of protein in the feed included shrimp meal, fishmeal, wheat flour, and chicken meal. The main sources of lipids in the feed were dissolved pulp, fish oil, squid paste, soy oil, and soy lecithin (Table 1). Specific formulations of premixes are shown in Table S1.

**Table 1:** Ingredients and proximate composition of experimental diets (%, DM basis).

Item	Treatments^1^							
	LFD	LF200	LF400	LF800	HFD	HF200	HF400	HF800
**Ingredients**								
Fermented soybean meal	4.69	4.69	4.69	4.69	0.00	0.00	0.00	0.00
Beer yeast	0.00	0.00	0.00	0.00	4.69	4.69	4.69	4.69
Yeast	3.13	3.13	3.13	3.13	0.00	0.00	0.00	0.00
Ca(H_2_PO_4_)_2_	1.75	1.75	1.75	1.75	1.75	1.75	1.75	1.75
Fish soluble	3.50	3.50	3.50	3.50	5.50	5.50	5.50	5.50
Shrimp meal	6.25	6.25	6.25	6.25	0.00	0.00	0.00	0.00
Antarctic krill meal	0.00	0.00	0.00	0.00	2.50	2.50	2.50	2.50
Squid paste	0.88	0.88	0.88	0.88	1.38	1.38	1.38	1.38
Fishmeal	25.00	25.00	25.00	25.00	29.38	29.38	29.38	29.38
Chicken meal	12.50	12.50	12.50	12.50	14.38	14.38	14.38	14.38
Dephenol cottonseed protein	0.00	0.00	0.00	0.00	4.78	4.78	4.78	4.78
Puffed soybean flour	0.00	0.00	0.00	0.00	10.63	10.63	10.63	10.63
Soybean meal	18.28	18.28	18.28	18.28	0.00	0.00	0.00	0.00
Wheat flour	17.50	17.50	17.50	17.50	17.50	17.50	17.50	17.50
Soybean phospholipid	1.75	1.75	1.75	1.75	2.75	2.75	2.75	2.75
Vitamin and mineral premix^2^	4.78	4.78	4.78	4.78	4.78	4.78	4.78	4.78
Bile acid, mg/kg	0.00	200.00	400.00	800.00	0.00	200.00	400.00	800.00
Total	100.00	100.00	100.00	100.00	100.00	100.00	100.00	100.00
**Proximate composition**								
DM	92.32	92.30	92.36	92.29	92.27	92.37	92.26	92.52
CP	45.32	45.29	45.17	45.12	45.38	45.25	45.15	45.21
CL	7.13	7.31	7.26	7.22	11.30	11.21	11.28	11.15
OM	91.68	92.37	92.14	92.47	91.55	92.42	91.56	91.62

DM = dry matter; OM = organic matter; CP = crude protein; CL = crude lipid.

1 LFD, LF200, LF400, and LF800 represent 7% lipid with 0, 200, 400, and 800 mg/kg BA, respectively; HFD, HF200, HF400, and HF800 represent 11% lipid with 0, 200, 400, and 800 mg/kg BA, respectively.

2 All raw materials and premixes were provided by Grobest Group Holdings Ltd. (CN), Fuzhou, Fujian, China. Specific formulations of premixes are shown in Table S1.

### 2.3 Feed preparation

Feed ingredients were ground and sieved through an 80-mesh screen, accurately weighed, and blended according to the experimental formulation. After the sequential addition of pre-mixed squid paste, soy lecithin, and fish-soluble extract, the mixture was re-sieved (80-mesh) and homogenized in a mixer for 15 min. Calcium dihydrogen phosphate and a controlled volume of water were then incorporated, followed by pelleting using an F-75 granulator (Huagong Optoelectronic Technology Co., Guangzhou, Guangdong, China). Pellets (1.0−1.5 mm diameter) were air-dried, vacuum-sealed in ziplock bags, and stored at −20 °C.

### 2.4 Experimental conditions and feeding trial

A total of 1280 shrimp (0.52 ± 0.01 g) were randomly assigned to 32 tanks (0.3 m^3^). The water temperature was maintained at 28 to 30 °C, salinity 6%–7%, the pH value hovered in the ideal range of 7.8 to 8.2, and the ammonia level was kept below 0.03 mg/L throughout the 8-week experiment. *L. vannamei* was fed at 8:00, 11:00, 17:00, and 21:00 every day, and feeding was stopped when satiety was evident. The remaining feed was removed with a siphon after the meal to assess feeding efficiency. Feeding rates were adjusted according to weather conditions and feeding effectiveness, while shrimp molting and growth were monitored daily.

### 2.5 Sample collection

Before sampling, shrimp were fasted overnight. The total weight and population of shrimp per tank were documented. For analytical purposes, shrimp were randomly allocated as follows: five for whole-body compositional analysis, eight for enzymatic assays, four for intestinal histology, and eight for intestinal microbiota profiling. Hemolymph was collected via aspiration from the fourth abdominal segment using a sterile 1-mL syringe and immediately centrifuged (3000 × *g*, 4 °C, 5 min) to isolate hemocytes. Hepatopancreas, intestinal, and muscle tissues were preserved in RNAlater (AM7021, Thermo Fisher Scientific Inc., Waltham, MA, USA) and stored at −80 °C until analysis. The body composition (moisture, crude protein [CP], and crude lipid [CL]) of feed and shrimp tissues was analyzed using AOAC (2005). Moisture content was quantified by gravimetric oven-drying at 105 °C (method 926.08) using an oven (DHG-9240 A, Shanghai Jingruo Company, Shanghai, China). Crude protein was analyzed via nitrogen determination (Organic Carbon/Nitrogen Analyzer, Primacs SNC100, Skalar Analytical B.V., Breda, North Brabant, the Netherlands) with a 6.25 conversion factor (method 968.06). Ash content analysis was performed by heating the sample in a muffle furnace (High-temperature resistance furnace, SX-8-10, Beijing Kaixing Demao Instrument Equipment Co., Ltd., Beijing, China) at 550 °C for 8 h (method 942.05). Organic matter (OM) content was calculated by subtracting the ash content from the dry matter (DM) content. Crude lipid was extracted using an XT15i solvent extractor (Ankom Technology Corp., Macedon, New York, USA) with petroleum ether, followed by gravimetric quantification (method 922.06).

### 2.6 Sample determination

#### 2.6.1 Biochemical indicators and enzyme activity analysis

Hepatopancreatic and hemolymphatic malondialdehyde (MDA; A003-1-2), triglycerides (TG; A110-1-1), low-density lipoprotein cholesterol (LDL-C; A113-1-1), high-density lipoprotein cholesterol (HDL-C; A112-1-1), non-esterified fatty acid (NEFA; A042-2-1), total cholesterol (T-CHO; A111-1-1), and total bile acids (TBA; E003-2-1) levels were quantified using standardized assay kits (Nanjing Jiancheng Bioengineering Institute, Nanjing, Jiangsu, China), following the manufacturer's protocols. Following centrifugation, the supernatant was collected and assayed at 550 nm with a UV-VIS spectrophotometer (UV–5800PC, Shanghai Yuanxi Instrument Co., Ltd., Shanghai, China).

#### 2.6.2 Histological section analysis and oil-red O staining analyses

Shrimp intestine samples were graded, dehydrated with ethanol, equilibrated with xylene, and fixed in Bouin's solution for more than 24 h before being embedded in paraffin wax as described previously (Xu et al., 2022c). Paraffin-embedded midgut tissues were cut into 4-μm thick sections using a Leica Jung RM 2016 rotary slicer (Leica Microsystems, Wetzlar, Hesse, Germany), followed by hematoxylin-eosin staining, and visualized under an ECLIPSE 90i inverted microscope (Nikon Corp., Tokyo, Japan) at 400× magnification. For oil-red O staining, the sections were made as described in Xu et al. (2022a). Hepatopancreas samples were stained with oil-red O to visualize lipid droplets (LDs; red) under an ECLIPSE 90i microscope at 400× magnification. Hepatopancreatic samples exhibiting > 30% cellular lysis were excluded from quantitative analysis. Subsequently, the integrated optical density (IOD) of stained areas was quantified using ImageJ software (v1.43).

#### 2.6.3 Transmission electron microscopy (TEM) and immunofluorescence analysis

Based on preliminary feeding trial results, 0 and 400 mg/kg BA were selected for subsequent trials. Focusing on these two doses enabled in-depth electron microscopy and microbiota analysis, adhering to experimental optimization principles. The intestines of fresh shrimp were removed and left in a 2.5% glutaraldehyde solution for 24 h. The sections were fixed with 1% OsO_4_ for 2 h at room temperature and protected from light, and then bleached with 0.1 malonate phosphate buffer (MPB) in ethanol after gradual dehydration, bleached with acetone, and finally embedded in resin. After being put on a copper grid, the ultrathin sections (60–80 nm) were dyed with 2% alcohol-saturated uranyl acetate and washed with distilled water. Lead citrate staining was applied for 8 min. Using a Transmission Electron Microscope (HT7700, Hitachi High-Tech Corp., Tokyo, Japan), the ultrathin sections were screened and examined, and seven different sites were measured in each section to measure the height and density of villi per unit length. According to the previously described method (Liang et al., 2016), paraffin sections obtained from the above histological section were incubated with anti-glutathione peroxidase 4 rabbit pAb (GB114327-100, Servicebio Technology Co., Ltd., Wuhan, Hubei, China) at 37 °C, followed by immunofluorescence analysis. Total nuclei were stained with blue 4′,6-diamidino-2-phenylindole (DAPI), and anti-glutathione peroxidase 4 nuclei were stained with red. The double-stained sections were analyzed using ImageJ software (v1.43).

#### 2.6.4 Real-time quantitative PCR

Total RNA was isolated from shrimp intestine and hepatopancreas tissues using the TransZol Up Plus RNA Kit (TransZol Up Plus, TransGen Biotech Co., Ltd., Beijing, China). First-strand complementary DNA (cDNA) synthesis was carried out with the Evo M-MLV RT Kit (AG11728, Accurate Biotechnology [Hunan] Co., Ltd., Changsha, Hunan, China), incorporating a gDNA Eraser step to eliminate genomic DNA contamination. Primer sequences are provided in Table S2. The RT-qPCR was performed on a LightCycler 480 II system (Real-time PCR System, LightCycler 480 II, Roche Diagnostics, Basel, Switzerland) utilizing SYBR Green Premix Pro Taq HS (qPCR Kit, AG11702, Accurate Biotechnology [Hunan] Co., Ltd., Changsha, Hunan, China). Thermal cycling denaturation conditions are referenced to Qu et al. (2024) with a cycle number of 40, annealing temperature adjusted depending on the primer, and final cooling 4 °C. The *ef-1α* gene served as the endogenous reference. The full names of the abbreviations of all genes can be found in Table S3. Relative gene expression was calculated using the 2^−ΔΔCt^ method (Livak and Schmittgen, 2001).

### 2.7 Intestinal microbiota analysis

Intestinal microbiota sequencing was performed by Gene Denovo Biotechnology Co. (Guangzhou, Guangdong, China). Microbial genomic DNA was extracted from intestinal samples using the HiPure Soil DNA Kit (Magen Biotechnology Co., Ltd., Guangzhou, Guangdong, China), following the manufacturer's protocol. The V3–V4 hypervariable regions of the bacterial 16 S rRNA gene were amplified using primers 341 F (5′-CCTACGGGNGGCWGCAG-3′) and 806 R (5′-GGACTACHVGGGTATCTAAT-3′). PCR amplicons were resolved on a 2% agarose gel, purified with the AxyPrep DNA Gel Extraction Kit (Axygen Biosciences, Union City, CA, USA), and quantified using an ABI StepOnePlus Real-Time PCR System (Thermo Fisher Scientific Inc., Waltham, MA, USA).

Post-sequencing, raw reads were processed using the following criteria: Removal of reads containing > 10% ambiguous bases (N) or with > 80% of bases exhibiting a Phred quality score (Q-score) ≤ 20; Paired-end read merging via FLASH v1.2.11 (Magoč and Salzberg, 2011) with default parameters (min. overlap: 10 bp, max. mismatch density: 0.25). Sequencing reads were quality-filtered and dereplicated, followed by chimera removal using the UCHIME algorithm. Clean reads were clustered into operational taxonomic units (OTUs) at ≥ 97% sequence similarity via UPARSE. Representative sequences from each OTU were taxonomically classified using the RDP classifier against the SILVA SSU database (v138) with a Naive Bayesian assignment algorithm (confidence threshold: 0.8).

Alpha diversity metrics (Chao1, Simpson, and Shannon indices) were calculated to assess microbial community richness and diversity. For biomarker discovery, linear discriminant analysis (LDA) effect size (LEfSe) was performed (LDA score [log_10_] > 2.0, *P* < 0.05). Beta diversity analysis involved sequence alignment with MUSCLE (v3.8.31), followed by weighted/unweighted UniFrac distance matrix computation (GUniFrac R package v1.7). Community structure variation was visualized via principal coordinates analysis (PCoA) based on Bray–Curtis dissimilarity (*ggplot2* v3.4.0).

### 2.8 Calculation formulas

$$\text{Weight}\;\text{gain}\;\text{rate}\;\left(\text{WG},\%\right)=100\times \{\left[\mathrm{Final}\;\mathrm{body}\;\mathrm{weight}\;(\mathrm{FBW})–\mathrm{Initial}\;\mathrm{body}\;\mathrm{weight}\;(\mathrm{IBW})\right]/\mathrm{IBW}\};$$

$$\text{Specific}\;\text{growth}\;\text{rate}\;\left(\text{SGR},\%/d\right)=100\times (\ln\text{FBW}–\mathrm{lnIBW})/\text{Number}\;\text{of}\;\text{experimental}\;\text{days}$$

$$\text{Survival}\;\left(\%\right)=(\text{Number}\;\text{of}\;\text{surviving}\;\text{individuals}/\text{Initial}\;\text{number}\;\text{of}\;\text{individuals) × 100;}$$

$$\text{IBW}\;\left(g\right)=\mathrm{Total}\;\mathrm{IBW}/\mathrm{Initial}\;\mathrm{number}\;\mathrm{of}\;\mathrm{individuals}\text{;}$$

$$\text{FBW}\;\left(g\right)=\mathrm{Total}\;\text{FBW}/\text{Number}\;\text{of}\;\text{surviving}\;\text{individuas;}$$

Feedconversionratio(FCR)=Dryfeedintake/Wetweightgain;

$$\text{Average}\;\text{daily}\;\text{feed}\;\text{intake}\;\left(\text{ADFI; g/d}\right)=\text{Feed}\;\text{consumed}\left(g\right)/\text{Number}\;\text{of}\;\text{experimental}\;\text{days;}$$

$$\text{Average}\;\text{daily}\;\text{gain}\;\left(\text{ADG; g/d}\right)=\left(\text{FBW}\;–\;\text{IBW}\right)/\text{Number}\;\text{of}\;\text{experimental}\;\text{days.}$$

### 2.9 Statistical analysis

All data were statistically validated using a two-way analysis of variance (two-way ANOVA). Before analysis, the normality and homogeneity of variance of the data were verified using the Shapiro–Wilk test and Levene's test, respectively. Statistical significance is defined as *P* < 0.05. The analysis was conducted in SPSS 22.0 (version 22.0, IBM Corp., Armonk, NY, USA), and post hoc pairwise comparisons were performed using Tukey's HSD test. Results are presented as means with standard error of the mean (SEM). Additionally, regression analysis was employed to assess linear and quadratic trends in the effects of different BA levels.

The linear regression model is as follows:

$$Y=\beta_{0}+\beta_{1}X+\varepsilon \text{;}$$

The quadratic regression model is as follows:

$$Y=\beta_{0}+\beta_{1}X+\beta_{2}X^{2}+\varepsilon \text{,}$$

where *Y* is the dependent variable; *X* is the independent variable (e.g., BA levels); *β*_0_ is the intercept, representing the predicted value of *Y* when *X* = 0; *β*_1_ is the Slope, indicating the change in *Y* for each unit increase in *X*; *β*_2_ is the coefficient of the quadratic term; *X*^*2*^ is the squared term of the independent variable, capturing the quadratic trend; *ε* is the error term, representing random variation not explained by the model.

The mathematical model for two-way ANOVA is represented as:

$$Y_{ijk}=\mu +\alpha_{i}+\beta j+{\left(\alpha \beta \right)}_{ij}+\varepsilon_{ijk}\text{,}$$

where *Y_ijk_* is the observation for the *k*-th replicate in the *i*-th lipid level and *j*-th bile acid level; *μ* is the overall mean across all groups; *α_i_* is the effect of the *i*-th lipid level (deviation from the grand mean); *β_j_* is the effect of the *j*-th bile acid level (deviation from the grand mean); (*αβ*)*_ij_* is the interaction effect between the *i*-th lipid level and the *j*-th bile acid level; *ε*_*ijk*_ is the random error term, which is assumed to be normally distributed with a mean of 0 and variance σ^2^.

## 3 Results

### 3.1 Growth performance and feed utilization

Dietary BA supplementation on growth performance and feed utilization of shrimp fed different lipid levels diets are shown in Table 2. The addition of BA significantly improved the FBW, WGR, and ADFI of shrimp (*P* < 0.05). The FBW (*P* = 0.008) and WGR (*P* = 0.019) of shrimp fed diets containing 400 mg/kg BA were significantly higher than those fed diets containing 0 or 200 mg/kg of BA. The FBW (*P* = 0.003), WGR (*P* = 0.005), and ADFI (*P* < 0.001) were increased linearly with dietary BA supplementation levels. Shrimp fed high-fat diets exhibited a significantly elevated FCR compared to those receiving low-fat diets (*P* = 0.047). Shrimp fed low-fat diets exhibited a significantly higher SGR compared to those fed high-fat diets (*P* = 0.039). The SR, FBW, IBW, WGR, FCR, SGR, ADFI, and ADG of shrimp exhibited no interactions between dietary lipid and BA levels (*P* > 0.05).

**Table 2:** Effects of bile acids (BA) on growth and physical parameters of *Litopenaeus vannamei* fed diets with different lipid levels.

Items	SR, %	FBW, g	IBW, g	FCR	WGR, %	SGR, %/d	ADFI, g/d	ADG, g/d
**Treatments**^1^								
LFD	93.75	9.95^B^	0.52	1.34^b^	1786.28^B^	5.21^a^	0.22^B^	0.16
LF200	98.75	10.02^B^	0.52	1.34^b^	1807.39^B^	5.22^a^	0.22^B^	0.17
LF400	91.87	10.84^A^	0.52	1.42^b^	1987.90^A^	5.23^a^	0.24^A^	0.17
LF800	91.25	10.72^AB^	0.53	1.46^b^	1943.03^A^	5.17^a^	0.24^A^	0.16
HFD	92.50	9.66^B^	0.53	1.47^a^	1748.89^B^	5.06^b^	0.22^B^	0.15
HF200	80.62	9.66^B^	0.52	1.73^a^	1755.39^B^	4.85^b^	0.22^B^	0.14
HF400	94.37	10.51^A^	0.54	1.40^a^	1859.56^A^	5.18^b^	0.23^A^	0.17
HF800	94.37	10.30^AB^	0.52	1.43^a^	1883.41^A^	5.18^b^	0.23^A^	0.16
SEM	0.197	0.038	0.388	0.052	0.067	0.108	0.001	0.265
***P*-value**^2^								
BA	0.810	0.008	0.450	0.350	0.019	0.307	<0.001	0.359
Linear	0.851	0.003	0.270	0.953	0.005	0.331	<0.001	0.271
Quadratic	0.602	0.609	0.497	0.405	0.764	0.607	0.333	0.900
Lipid	0.256	0.085	0.295	0.047	0.100	0.039	0.599	0.122
BA × Lipid	0.058	0.996	0.298	0.050	0.854	0.202	0.317	0.330

SEM = standard error of the mean; SR = survival rate; FBW = final body weight; IBW = initial body weight; WGR = weight gain rate; SGR = specific growth rate; FCR = feed conversion ratio; ADFI = average daily feed intake; ADG = average daily gain.

1 LFD, LF200, LF400, and LF800 represent 7% lipid with 0, 200, 400, and 800 mg/kg BA, respectively; HFD, HF200, HF400, and HF800 represent 11% lipid with 0, 200, 400, and 800 mg/kg BA, respectively.

2 Statistical significance was set at *P* < 0.05 (*n* = 4). Significant differences between dietary BA levels are indicated by different superscript capital letters within columns (*P* < 0.05), while different superscript lowercase letters denote significant differences between dietary lipid levels (*P* < 0.05), BA × Lipid indicates the BA level and lipid interaction level (*P* < 0.05). The *P* values of linear and quadratic were the differences among groups.

### 3.2 The body composition of whole shrimp

Dietary BA supplementation on the body composition of whole shrimp fed diets with different lipid levels are shown in Table 3. The CL content of the shrimp fed the 400 mg/kg BA diet was markedly lower than that of shrimp fed the 200 mg/kg BA diet (*P* = 0.021). Shrimp fed high-fat diets exhibited significantly elevated whole-body CL content compared to those maintained on low-fat diets (*P* < 0.001). There was an interaction between dietary lipid levels and BA in terms of the crude fat content of shrimp (*P* < 0.05).

**Table 3:** Effects of bile acids (BAs) on whole shrimp body composition of *Litopenaeus vannamei* fed diets with different lipid levels (%, based on dry weight).

Item	Moisture	Crude lipid	Crude protein	Crude ash
**Treatments**^1^				
LFD	76.7	0.028^ABb^	14.34	4.68
LF200	79.2	0.034^Ab^	14.38	4.44
LF400	78.7	0.025^Bb^	14.37	4.56
LF800	78.7	0.030^ABb^	14.41	4.67
HFD	78.0	0.033^ABa^	14.26	4.45
HF200	79.2	0.031^Aa^	14.33	4.38
HF400	77.0	0.035^Ba^	14.38	4.51
HF800	78.0	0.031^ABa^	14.32	4.68
SEM	0.66	0.000	0.480	0.273
***P*-value**^2^				
BA	0.418	0.021	0.418	0.109
Linear	0.114	0.164	0.179	0.169
Quadratic	0.651	0.436	0.326	0.052
Lipid	0.547	<0.001	0.148	0.227
BA × Lipid	0.636	<0.001	0.682	0.708

SEM = standard error of the mean.

1 LFD, LF200, LF400, and LF800 represent 7% lipid with 0, 200, 400, and 800 mg/kg BA, respectively; HFD, HF200, HF400, and HF800 represent 11% lipid with 0, 200, 400, and 800 mg/kg BA, respectively.

2 Statistical significance was set at *P* < 0.05 (*n* = 4). Significant differences between dietary BA levels are indicated by different superscript capital letters within columns (*P* < 0.05), while different superscript lowercase letters denote significant differences between dietary lipid levels (*P* < 0.05), BA × Lipid indicates the BA level and lipid interaction level (*P* < 0.05). The *P* values of linear and quadratic were the differences among groups.

### 3.3 Analysis of hemolymph and hepatopancreas enzyme activity

The lipid metabolism-related parameters of hemolymph and hepatopancreas in shrimp are presented in Table 4. In hemolymph, the LDL-C content was significantly higher in shrimp fed the 800 mg/kg BA diet than those fed other diets, and levels in shrimp fed 200 or 400 mg/kg BA were also higher than in those fed the 0 mg/kg diet (*P* < 0.001). Shrimp fed high-fat diet exhibited significantly higher LDL-C levels than those fed low-fat diet, and LDL-C increased linearly with BA supplementation (*P* < 0.001). The HDL-C content was markedly higher in shrimp fed 200 mg/kg BA than those fed other diets (*P* = 0.003), showing a quadratic trend that peaked at 200 mg/kg (*P* = 0.013). The TG content was significantly lower in shrimp fed 400 or 800 mg/kg BA than in those fed 0 or 200 mg/kg BA, and was also lower in the 200 mg/kg group than in the 0 mg/kg group (*P* < 0.001). Shrimp fed high-fat diet exhibited significantly reduced TG content compared to those on low-fat diet (*P* < 0.001), and TG levels showed significant linear and quadratic responses to BA, decreasing sharply at lower doses before plateauing above 400 mg/kg (*P* < 0.001 and *P* = 0.002, respectively). The T-CHO content was markedly higher in shrimp fed 200 and 800 mg/kg BA than in those fed 0 or 400 mg/kg BA, was higher in the high-fat group than in the low-fat group, and increased linearly with BA supplementation (*P* < 0.001).

**Table 4:** Effects of bile acids (BAs) on hemolymph biochemistry of *Litopenaeus vannamei* under different dietary lipid levels (mmol/L).

Item	LDL-C	HDL-C	TG	T-CHO	MDA
**Treatments**^1^					
LFD	0.24^Cb^	0.21^B^	1.51^Aa^	1.04^Bb^	4.73^b^
LF200	0.28^Bb^	0.22^A^	1.02^Ba^	1.16^Ab^	4.41^b^
LF400	0.29^Bb^	0.18^B^	0.77^Ca^	1.27^Bb^	4.36^b^
LF800	0.32^Ab^	0.15^B^	0.56^Ca^	1.22^Ab^	5.90^b^
HFD	0.27^Ca^	0.11^B^	0.75^Ab^	1.32^Ba^	5.53^a^
HF200	0.436^Ba^	0.23^A^	0.64^Bb^	1.79^Aa^	5.59^a^
HF400	0.34^Ba^	0.16^B^	0.40^Cb^	1.26^Ba^	6.22^a^
HF800	0.52^Aa^	0.19^B^	0.57^Cb^	1.87^Aa^	4.52^a^
SEM	0.000	0.000	0.000	0.000	0.000
***P*-value**^2^					
BA	<0.001	0.003	<0.001	<0.001	0.511
Linear	<0.001	0.682	<0.001	<0.001	0.405
Quadratic	0.759	0.013	0.002	0.862	0.851
Lipid	<0.001	0.113	<0.001	<0.001	<0.001
BA × Lipid	0.001	0.001	<0.001	<0.001	<0.001

SEM = standard error of the mean; LDL-C = low-density lipoprotein; TG = triglyceride; T-CHO = total cholesterol; NEFA = non-esterified fatty acid; MDA = malondialdehyde.

1 LFD, LF200, LF400, and LF800 represent 7% lipid with 0, 200, 400, and 800 mg/kg BA, respectively; HFD, HF200, HF400, and HF800 represent 11% lipid with 0, 200, 400, and 800 mg/kg BA, respectively.

2 Statistical significance was set at *P* < 0.05 (*n* = 4). Significant differences between dietary BA levels are indicated by different superscript capital letters within columns (*P* < 0.05), while different superscript lowercase letters denote significant differences between dietary lipid levels (*P* < 0.05), BA × Lipid indicates the BA level and lipid interaction level (*P* < 0.05). The *P* values of linear and quadratic were the differences among groups.

As shown in Table 5, in hepatopancreas, the non-esterified fatty acid (NEFA) content was markedly lower in shrimp fed 400 or 800 mg/kg BA than in those fed 200 mg/kg BA, while shrimp fed 0 mg/kg BA showed lower content than those fed 800 mg/kg BA (*P* < 0.001). Shrimp fed high-fat diet exhibited significantly higher NEFA levels than those on low-fat diet, and NEFA showed a quadratic trend, peaking at 200 mg/kg BA (*P* < 0.001). The T-CHO content was markedly higher in shrimp fed 200 mg/kg BA than in those fed 0 or 400 mg/kg BA, while shrimp fed 800 mg/kg BA showed lower content than those fed 0 or 400 mg/kg BA (*P* < 0.001). The high-fat group had a markedly higher T-CHO content than the low-fat group, and T-CHO showed significant linear and quadratic responses to BA, peaking at 200 mg/kg and reaching the lowest level at 800 mg/kg (*P* < 0.001). The TG content was markedly lower in shrimp fed 200 mg/kg BA than in those fed 0 or 400 mg/kg BA, and was significantly lower in the 800 mg/kg group than in the 200 mg/kg group (*P* < 0.001). Shrimp fed high-fat diet exhibited significantly higher TG content than those on low-fat diet (*P* < 0.001), and TG showed significant linear and quadratic responses to BA, peaking at 0 and 400 mg/kg and reaching the lowest level at 800 mg/kg (*P* < 0.001 and *P* = 0.007, respectively). The LDL-C content was significantly lower in shrimp fed 400 mg/kg BA than in those fed 200 or 800 mg/kg BA (*P* = 0.011). Enzyme activity results in hemolymph and hepatopancreas suggest a significant interaction between dietary lipids and BA levels (*P* < 0.05).

**Table 5:** Effects of bile acids (BAs) on hepatopancreas biochemistry of *Litopenaeus vannamei* under different dietary lipid levels.

Item	TG, mmol/g prot	LDL-C, mmol/g prot	T-CHO, mmol/g prot	NEFA, μmol/g prot	MDA, mmol/g prot
**Treatments**^1^					
LFD	1.81^Ab^	0.46^AB^	0.95^Bb^	1.30^Cb^	12.91^Ab^
LF200	1.39^Bb^	0.56^A^	1.04^Ab^	1.64^Ab^	12.75^ABb^
LF400	2.17^Ab^	0.72^B^	0.75^Bb^	1.06^BCb^	10.26^Cb^
LF800	1.24^Cb^	0.87^A^	0.76^Cb^	2.32^Bb^	13.88^Bb^
HFD	2.82^Aa^	0.79^AB^	1.17^Ba^	2.39^Ca^	17.97^Aa^
HF200	2.19^Ba^	0.75^A^	1.65^Aa^	4.24^Aa^	17.41^ABa^
HF400	2.56^Aa^	0.45^B^	1.39^Ba^	3.10^BCa^	12.43^Ca^
HF800	1.39^Ca^	0.49^A^	0.92^Ca^	2.21^Ba^	15.00^Ba^
SEM	0.000	0.000	0.000	0.000	0.000
***P*-value**^2^					
BA	<0.001	0.011	<0.001	<0.001	<0.001
Linear	<0.001	0.258	<0.001	0.368	<0.001
Quadratic	0.007	0.081	<0.001	<0.001	<0.001
Lipid	<0.001	0.122	<0.001	<0.001	<0.001
BA × Lipid	0.001	<0.001	0.001	<0.001	<0.001

SEM,= standard error of the mean; TG = triglyceride; LDL-C = low-density lipoprotein; prot = protein; T-CHO = total cholesterol; NEFA = non-esterified fatty acid; MDA = malondialdehyde.

1 LFD, LF200, LF400, and LF800 represent 7% lipid with 0, 200, 400, and 800 mg/kg BA, respectively; HFD, HF200, HF400, and HF800 represent 11% lipid with 0, 200, 400, and 800 mg/kg BA, respectively.

2 Statistical significance was set at *P* < 0.05 (*n* = 4). Significant differences between dietary BA levels are indicated by different superscript capital letters within columns (*P* < 0.05), while different superscript lowercase letters denote significant differences between dietary lipid levels (*P* < 0.05), BA × Lipid indicates the BA level and lipid interaction level (*P* < 0.05). The *P* values of linear and quadratic were the differences among groups.

### 3.4 Lipid deposition in the hepatopancreas of shrimp

**Fig. 1:**
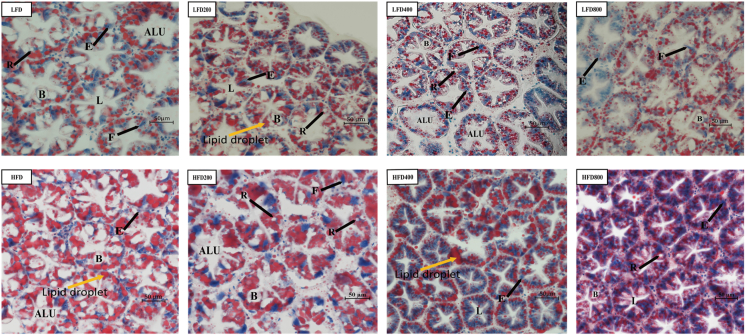
Effects of bile acids (BAs) added to diets with different lipid levels on hepatopancreatic lipid accumulation in shrimp. The yellow arrow points to the lipid droplets. Scale bar, 50 μm. L = lumen; ALU = abnormal lumen; B = secretory cell; L = hepatic tubule; E = embryonic cell; F = fibrillazellen-cell; R = resorptive cell. LFD, LF200, LF400, and LF800 represent 7% lipid with 0, 200, 400, and 800 mg/kg BA, respectively; HFD, HF200, HF400, and HF800 represent 11% lipid with 0, 200, 400, and 800 mg/kg BA, respectively.

The histological staining of hepatopancreatic tissue with lipid droplets displays in Fig. 1A. Hepatopancreatic lipid deposition in shrimp decreased significantly with increasing dietary BA levels, with the lowest deposition observed in the 400 mg/kg group, which was significantly lower than that in all other groups (*P* < 0.001, Table 7). Shrimp fed high-fat diets exhibited significantly higher hepatopancreatic lipid droplet density compared to those receiving low-fat diets (*P* < 0.001). Further analysis revealed a significant interaction between dietary lipid levels and BA supplementation (*P* < 0.001).

**Table 6:** Effects of bile acids (BAs) on the diversity and abundance of the intestinal microbiota of *Litopenaeus vannamei* fed diets with different lipid levels.

Item	Sobs index	Shannon index	Simpson index	Chao1 index	ACE index
**Treatments**^1^					
LFD	174.50^Bb^	2.00^Bb^	0.50^Bb^	220.74	213.36
LF400	175.75^Ab^	2.36^Ab^	0.59^Ab^	215.63	218.61
HFD	154.75^Ba^	2.36^Ba^	0.63^Ba^	215.41	212.35
HF400	259.25^Aa^	3.78^Aa^	0.78^Aa^	278.74	290.86
SEM	0.001	0.000	0.013	0.132	0.055
***P*-value**^2^					
BA	0.003	0.001	0.034	0.180	0.067
Lipid level	0.041	0.001	0.007	0.183	0.113
BA × Lipid	0.003	0.023	0.511	0.120	0.104

SEM = standard error of the mean.

1 LFD, and LF400 represent 7% lipid with 0 and 400 mg/kg BA, respectively; HFD, and HF400, represent 11% lipid with 0 and 400 mg/kg BA, respectively.

2 Statistical significance was set at *P* < 0.05 (*n* = 4). Significant differences between dietary BA levels are indicated by different superscript capital letters within columns (*P* < 0.05), while different superscript lowercase letters denote significant differences between dietary lipid levels (*P* < 0.05), BA × Lipid indicates the BA level and lipid interaction level (*P* < 0.05). The *P* values of linear and quadratic were the differences among groups.

**Table 7:** Hepatopancreatic lipid droplet area in shrimp (%).

Items	Lipid droplet area
**Treatments**^1^	
LFD	10.12^Ab^
LF200	13.20^Bb^
LF400	6.89^Db^
LF800	11.46^Cb^
HFD	30.28^Aa^
HF200	25.16^Ba^
HF400	10.54^Da^
HF800	11.21^Ca^
SEM	0.000
***P*-value**^2^	
BA	<0.001
Linear	<0.001
Quadratic	<0.001
Lipid	<0.001
BA × Lipid	<0.001

BA = bile acids; SEM = standard error of the mean.

1 LFD, LF200, LF400, and LF800 represent 7% lipid with 0, 200, 400, and 800 mg/kg BA, respectively; HFD, HF200, HF400, and HF800 represent 11% lipid with 0, 200, 400, and 800 mg/kg BA, respectively.

2 Statistical significance was set at *P* < 0.05 (*n* = 4). Significant differences between dietary BA levels are indicated by different superscript capital letters within columns (*P* < 0.05), while different superscript lowercase letters denote significant differences between dietary lipid levels (*P* < 0.05), BA × Lipid indicates the BA level and lipid interaction level (*P* < 0.05). The *P* values of linear and quadratic were the differences among groups.

Moreover, the hepatopancreas in the HF400 and HF800 groups exhibited a well-organized tubular structure typical of *L. vannamei*, characterized by tightly arranged hepatopancreatic tubules with star-shaped lumina and abundant R-cells. In contrast, the low-fat groups (LFD, LF200, and LF400) showed swollen B-cells, lighter coloration, and disorganized tubule structure. Notably, these groups displayed a loss of normal luminal architecture, blurred tubular outlines, and reduced R-cell populations.

### 3.5 The expression of lipid metabolism of hepatopancreas and intestine in shrimp

**Fig. 2:**
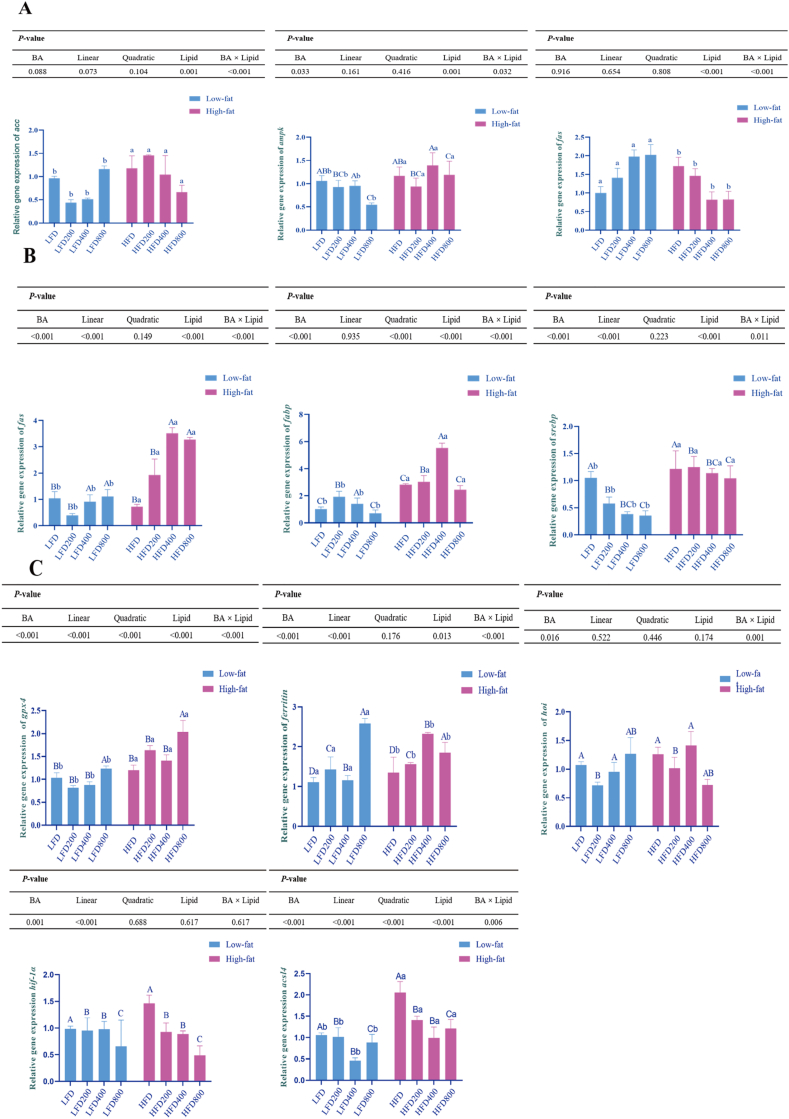
Effects of dietary bile acid (BAs) on the expression of lipid metabolism- and ferroptosis-related genes in the hepatopancreas and intestine of shrimp fed diets with different lipid levels. (A) Effect of bile acid on lipid metabolism-related genes in the hepatopancreas of shrimp fed different lipid levels diets. (B) Effect of bile acid on lipid metabolism-related genes in the intestine of shrimp fed different lipid levels diets. (C) Effect of bile acid on ferroptosis-related genes in the hepatopancreas of shrimp fed different lipid levels diets. LFD, LF200, LF400, and LF800 represent 7% lipid with 0, 200, 400, and 800 mg/kg BA, respectively; HFD, HF200, HF400, and HF800 represent 11% lipid with 0, 200, 400, and 800 mg/kg BA, respectively. SEM = standard error of the mean. Different capital letters in the figure indicate significant differences (*P* < 0.05) among BA levels, while different lowercase letters denote significant differences among dietary lipid levels (*P* < 0.05); *n* = 4.

The lipid metabolism-related genes of the hepatopancreas in shrimp are presented in Fig. 2A. Compared to those maintained on low-fat diet, shrimp fed high-fat diet exhibited significantly elevated *acc* gene expression (*P* = 0.001), upregulated *ampk* expression (*P* = 0.001), and markedly reduced *fas* expression (*P* < 0.001) in the hepatopancreas. The gene expression of *ampk* in the hepatopancreas was significantly higher in shrimp fed the 400 mg/kg BA diet compared to those fed 200 or 800 mg/kg BA diet (*P* = 0.033). A notable interaction was found between dietary lipids and BA levels affecting the expression of *acc*, *ampk*, and *fas* in the hepatopancreas (*P* < 0.05).

The lipid metabolism-related genes of the intestine in shrimp are presented in Fig. 2B. In intestinal tissues, shrimp fed high-fat diet exhibited significantly elevated gene expression of *fas* and *fabp* compared to those maintained on low-fat diet, while the gene expression of *srebp* was also significantly increased (*P* < 0.001). Regarding dietary BA levels, the gene expression of *fas* was significantly higher in shrimp fed 400 or 800 mg/kg BA diet compared to those fed 0 or 200 mg/kg BA diet, and increased linearly with BA supplementation (*P* < 0.001). The *fabp* gene expression was significantly higher in shrimp fed 400 mg/kg BA compared to those fed other diets, and was also higher in those fed 200 mg/kg BA compared to those fed 0 or 800 mg/kg diet (*P* < 0.001), showing a quadratic trend that peaked at 200 mg/kg BA (*P* < 0.001). The gene expression of *srebp* decreased with the level of supplement BA addition (*P* < 0.001) and decreased linearly with BA supplementation (*P* < 0.001). A notable interaction was identified between dietary lipids and BA levels concerning the gene expression of *fas*, *fabp*, and *srebp* in the intestine (*P* < 0.05).

### 3.6 Ferroptosis indicators: lipid peroxides, immunofluorescence analysis, and gene expression

As shown in Table 4, Table 5, shrimp fed high-fat diet exhibited significantly elevated MDA levels in both hemolymph and hepatopancreas compared to those maintained on low-fat diet (*P* < 0.001). The MDA content in the hepatopancreas was significantly lower in shrimp fed the 400 mg/kg BA diets compared to all other dietary groups (*P* < 0.001). The MDA content in shrimp hepatopancreas showed significant linear and quadratic responses to BA supplementation, with levels decreasing progressively to reach minimal values at 400 mg/kg before showing slight elevation at 800 mg/kg (*P* < 0.001).

The ferroptosis-related genes of the hepatopancreas in shrimp are presented in Fig. 2C. The gene expression of *gpx4* in the hepatopancreas was significantly higher in shrimp fed the 800 mg/kg BA diet compared to all other dietary groups (*P* < 0.001). Shrimp fed high-fat diet also exhibited significantly elevated *gpx4* gene expression compared to those maintained on low-fat diet, and the expression showed significant linear and quadratic responses to BA supplementation, rising slightly at 200 mg/kg before increasing sharply to peak at 800 mg/kg (*P* < 0.001). The *fer* gene expression in the hepatopancreas increased with the level of dietary BA addition and increased linearly with BA supplementation (*P* < 0.001), but was significantly reduced in shrimp fed high-fat diets compared to those on low-fat diet (*P*= 0.013). The *hif-1α* gene expression in the hepatopancreas was significantly higher in shrimp fed 0 or 400 mg/kg BA diet compared to those fed the 200 mg/kg BA diet (*P* = 0.016). The *hif-1α* gene expression in the hepatopancreas decreased with increasing dietary BA level (*P* = 0.001) and decreased linearly with BA supplementation (*P* < 0.001). Similarly, the *acsl4* gene expressiondecreased with increasing dietary BA level and decreased linearly with BA supplementation, but was significantly elevated in shrimp fed high-fat diet compared to those on low-fat diet (*P* < 0.001). A notable interaction was observed between dietary lipids and BA levels regarding malondialdehyde and the expression of the ferroptosis-related genes *fer*, *hif-1α*, *gpx4*, and *acsl4* in shrimp (*P* < 0.05).

**Fig. 3:**
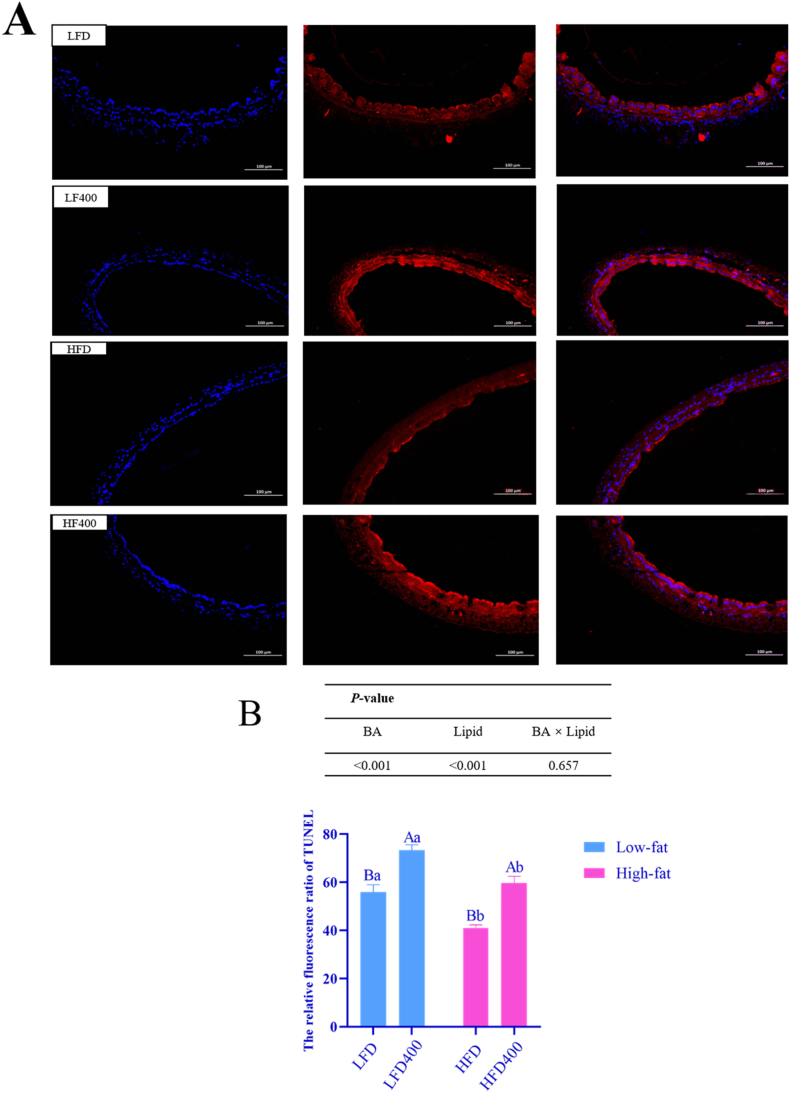
Effects of bile acids (BAs) supplementation in diets with different lipid levels on GPX4 protein immunofluorescence in the intestine of shrimp. (A) GPX4 immunofluorescence staining in the intestine. (B) Relative fluorescence intensity of GPX4. LFD and LF400 represent 7% lipid with 0 and 400 mg/kg BA, respectively; HFD and HF400, represent 11% lipid with 0 and 400 mg/kg BA, respectively; *n* = 4. Relative fluorescence intensity = Integrated density/Area. Different capital letters in the figure indicate significant differences (*P* < 0.05) among dietary BA levels, while different lowercase letters denote significant differences among dietary lipid levels (*P* < 0.05); *n* = 4.

As shown in Fig. 3A and B, the relative ratio of GPX4 immunofluorescence in the shrimp intestine was significantly higher in shrimp fed the 400 mg/kg BA diet compared to the 0 mg/kg BA diet, but was reduced in the high-fat group compared to the low-fat group (*P* < 0.001).

### 3.7 Morphological analysis of intestine

**Fig. 4:**
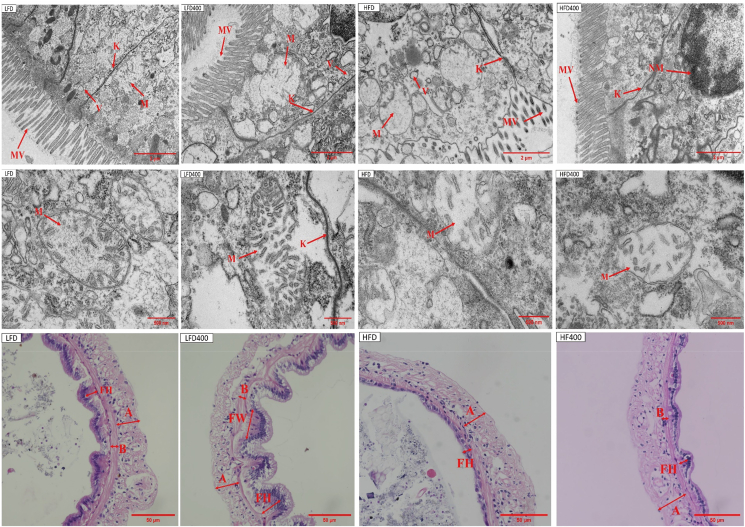
Effects of bile acids (BAs) added to feeds with different lipid levels on midgut morphology transmission electron microscopy (TEM) and paraffin sections of shrimp. Midgut samples were hematoxylin and eosin (H&E) staining. The scale represents 2 μm and 500 nm (TEM), and 50 μm H&E. LFD, LF200, LF400, and LF800 represent 7% lipid with 0, 200, 400, and 800 mg/kg BA, respectively; HFD, HF200, HF400, and HF800 represent 11% lipid with 0, 200, 400, and 800 mg/kg BA, respectively. K = cell tight junction; N = nucleus; NM = nuclear membrane; M = mitochondrion; MV = Microvilli vesicle; V = vesicle; FH = fold height; FW = fold width; A = basal thickness; B = muscle thickness.

The morphological analysis of the intestine in shrimp is presented in Fig. 4. The intestinal fold width and fold height were markedly higher in shrimp fed a diet containing 400 mg/kg BA compared to those fed 0 mg/kg BA (*P* < 0.001, Table 8). Conversely, in the high-fat group, the intestinal fold width, fold height, and microvilli height were all significantly lower than those in the low-fat group, and the intestinal muscle thickness was also markedly reduced (*P* < 0.001). The microvilli height in the intestine was markedly higher in shrimp fed the 400 mg/kg BA diet compared to those fed the 0 mg/kg BA diet (*P* < 0.001). A notable correlation was detected between dietary lipids and BA levels on intestinal fold width, fold height, muscle thickness, and microvilli height in shrimp (*P* < 0.05).

**Fig. 5:**
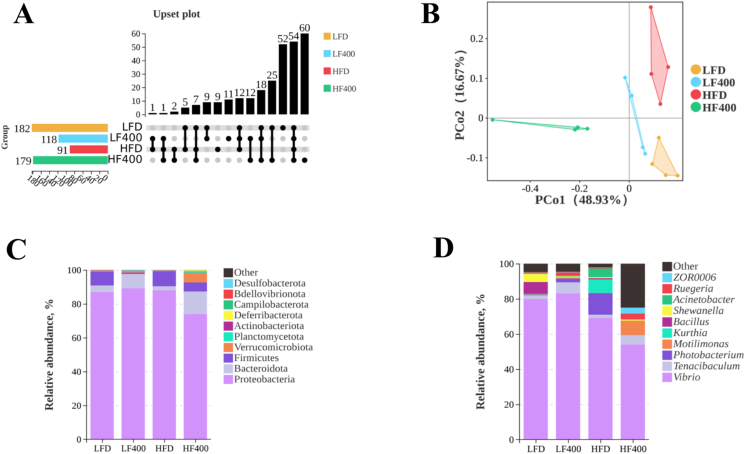
Effects of bile acids (BAs) added to diets with different lipid levels on the intestinal microbial composition of the shrimp. (A) Upset plots showing the species composition of the intestinal microbiota of shrimp in different groups. In the upset plot, differently colored bars represent distinct groups (left vertical axis), with their length indicating the total number of species per group. The intersection matrix (center)—composed of connected black dots and lines—visually depicts overlaps between groups: dots indicate a group's participation in a specific intersection, while blank spaces denote absence. The right-side bar plot displays the number of species unique to each combination (including exclusive and shared subsets). (B) Principal coordinates analysis (PCoA). Statistical significance was assessed by permutational multivariate analysis of variance (PERMANOVA; *P* = 0.001), a non-parametric method used to test the hypothesis that the centroids and dispersion of microbial communities are similar across groups. Intestinal microbiota composition at the phylum (C) and genus (D) level. LFD and LF400 represent 7% lipid with 0 and 400 mg/kg BA, respectively; HFD and HF400, represent 11% lipid with 0 and 400 mg/kg BA, respectively.

**Table 8:** Effects of dietary bile acids (BAs) on the intestinal morphology of *Litopenaeus vannamei* fed diets with different lipid levels.

Items	Fold width**, μm**	Fold height**, μm**	Basal thickness**, μm**	Muscle thickness**, μm**	Microvilli height**, μm**	Microvilli**, /μm**
**Treatments**^1^						
LFD	12.11^Ba^	16.07^Ba^	31.21	7.89^a^	1.60	20.75^B^
LF400	34.75^Aa^	29.35^Aa^	31.36	9.46^a^	1.64	21.25^A^
HFD	6.24^Bb^	6.38^Bb^	30.20	4.44^b^	0.74	10.75^B^
HF400	22.86^Ab^	11.33^Ab^	32.86	3.51^b^	1.35	24.75^A^
SEM	0.000	0.000	0.249	0.000	0.000	0.001
***P*-value**^2^						
BA	<0.001	<0.001	0.134	0.125	<0.001	0.002
Lipid level	<0.001	<0.001	0.783	<0.001	<0.001	0.107
BA × Lipid	<0.001	<0.001	0.179	<0.001	<0.001	0.004

SEM = standard error of the mean.

1 LFD, and LF400 represent 7% lipid with 0 and 400 mg/kg BA, respectively; HFD, and HF400, represent 11% lipid with 0 and 400 mg/kg BA, respectively.

2 Statistical significance was set at *P* < 0.05 (*n* = 4). Significant differences between dietary BA levels are indicated by different superscript capital letters within columns (*P* < 0.05), while different superscript lowercase letters denote significant differences between dietary lipid levels (*P* < 0.05), BA × Lipid indicates the BA level and lipid interaction level (*P* < 0.05). The *P* values of linear and quadratic were the differences among groups.

### 3.8 Intestinal microbiota

#### 3.8.1 Richness and diversity analysis of the intestinal microbiota

A total of 3,517,219 raw tags were churned out from the intestinal microbiome, tallying up to 109,913 per sample on average. Following a rigorous sequence analysis and a thorough filtering process, it was ended up with 3,226,707 viable tags, which came out to be roughly 100,834 per sample (Table S4). The mean sequence length was 697 bp (Table S5). More than 91.35% of high-quality effective tags were used in the OTU clustering process (Table S4). Based on the identified outliers in the principal coordinates analysis and effective ratio values (Table S4), four representative data points per experimental group were chosen for subsequent analysis. The selected samples included: LF1-2, LF1-4, LF1-6, and LF1-7 from the LFD group; LF3-2, LF3-3, LF3-4, and LF3-8 from the LF400 group; HF5-1, HF5-3, HF5-5, and HF5-7 from the HFD groups; and HF7-1, HF7-2, HF7-3, and HF7-7 from the HF400 group. Based on the Upset plot, it can be seen that the four groups collectively had 54 identical species at the genus level, with the lowest number of species in the intestinal microbiota of shrimp fed the HFD diet and the highest number of species in the intestinal microbiota of shrimp fed the LFD diet, and it is worth noting that there were 60 and 52 group-specific numbers of species for the HF400 and LFD, respectively (Fig. 5A). Alpha diversity analysis was carried out on LFD, LF400, HFD, and HF400 groups to study the effect of BA addition on shrimp intestinal microbiota at different lipid levels. Interestingly, the diversity coefficients of Sobs, Shannon, and Simpson of shrimp-fed diets supplemented with 400 mg/kg BA were significantly higher than those of shrimp-fed diets supplemented with 0 mg/kg BA (*P* < 0.05; Table 6). Shrimp fed high-fat diets exhibited significantly elevated Sobs, Shannon, and Simpson indices in their intestinal microbiota compared to those receiving low-fat diets (*P* < 0.05). Good's coverage for every sample surpassed 99.50%, suggesting reliable sequencing accuracy. Beta diversity analysis was demonstrated by pairwise sample similarity analysis based on Bray–Curtis distances between samples. The four groups were found to be separated by PCOA analysis plot (Fig. 5B). Anosim and Adonis analyses indicated significant differences in intestinal microbiota among the four groups, which was further confirmed by two non-parametric tests: analysis of similarity (Anosim, *R* = 0.589, *P* = 0.001) and multivariate variance equation analysis (Adonis, *R*^2^ = 0.570, *P* = 0.001; Fig. S1).

**Fig. 6:**
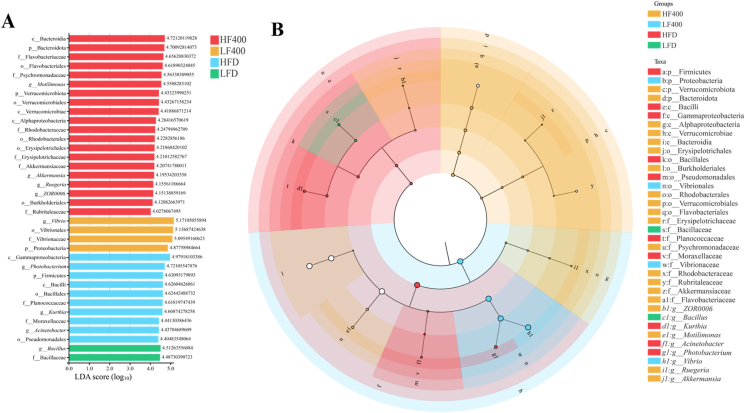
Effects of bile acids (BAs) added to diets with different lipid levels on the intestinal microbial composition of the shrimp. (A) Histogram of linear discriminant analysis (LDA) scores (log_10_) for taxa with significant differential abundance (LDA score > 4). Only the highest-scoring taxa per group are shown. (B) Cladogram illustrating the phylogenetic distribution of discriminant taxa. LFD and LF400 represent 7% lipid with 0 and 400 mg/kg BA, respectively; HFD and HF400, represent 11% lipid with 0 and 400 mg/kg BA, respectively. Red nodes, enriched in HF400 group; green nodes, enriched in HFD group; blue nodes, enriched in LFD group.

#### 3.8.2 Species compositional analysis of the intestinal microbiota

**Fig. 7:**
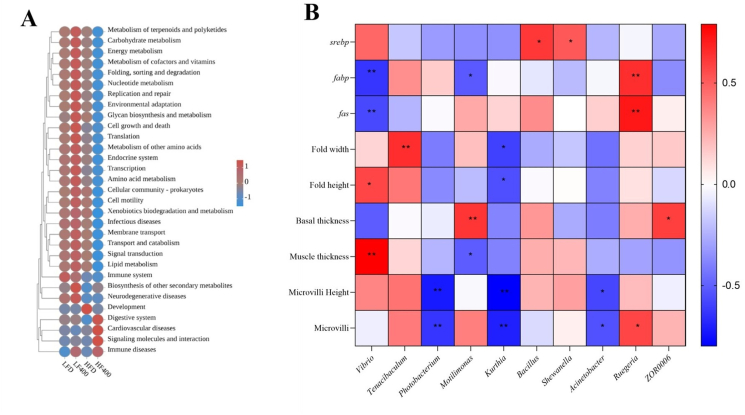
Analysis of microbial Kyoto Encyclopedia of Genes and Genomes (KEGG) functions and correlations with host indicators. (A) Heatmap of functional abundance of different groups obtained by clustering analysis of multiple KEGG functional abundances. (B) Correlation analysis between the top 10 dominant genus and gut-related indicators. A displays correlation coefficients through a color gradient, with red and blue, respectively, indicating high and low functional abundance levels. B specifically examines relationships between the top 10 dominant bacterial genera (X-axis) and both intestinal lipid metabolism genes and morphological parameters (Y-axis), where the color scale represents *R* values for positive (red) and negative (blue) correlations. LFD and LF400 represent 7% lipid with 0 and 400 mg/kg BA, respectively; HFD and HF400, represent 11% lipid with 0 and 400 mg/kg BA, respectively. ∗ indicates a highly significant correlation above 0.01 (two-tailed test) and ∗∗ indicates a significant correlation above 0.05 (two-tailed test).

In this study, the predominant phyla were Proteobacteria, Bacteroidota, Firmicutes, Verrucomicrobiota, and Planctomycetota, as shown in Fig. 5, Fig. 7. Notably, the abundance of Proteobacteria in the intestinal microbiota was significantly lower in shrimp fed the 400 mg/kg of BA diet compared to those fed the 0 mg/kg of BA diet (*P* = 0.034; Table 9), whereas the intestinal microbiota of shrimp fed a low-fat diet had a significantly higher abundance of Proteobacteria than those of shrimp fed high-fat diets (*P* = 0.015). The abundance of Bacteroidota and Verrucomicrobiota in the intestinal microbiota was significantly higher in shrimp fed the 400 mg/kg BA diet compared to those fed the 0 mg/kg BA diet (*P*< 0.001), Among them, Verrucomicrobiota and Planctomycetota of the intestinal microbiota of shrimp fed high-fat diet were markedly higher than those fed low-fat diet (*P* = 0.001; Fig. 6A). Figure 5D presents the relative abundance of intestinal microbiota at the genus level across experimental groups. The major bacterial genera of shrimp include *Vibrio*, *Tenacibaculum*, *Photobacterium*, *Motilimona*s, and *Kurthia*. To investigate the interactive effects of dietary lipid and BA on intestinal microbiota composition, the relative abundance of the ten dominant genera was analyzed (Table 9). The relative abundance of *Tenacibaculum*, *Motillmonas*, and *Ruegeria* significantly increased with the addition of BA to the diet (*P* < 0.05). Conversely, the relative abundance of *Kurthia* in the intestinal microbiota of shrimp significantly decreased with the addition of BA to the diet (*P* = 0.002). In addition, the abundance of *Vibrio* in the intestinal microbiota of shrimp fed high-fat diets was significantly lower than that of shrimp fed low-fat diets (*P* < 0.001). Whereas, the intestinal microbiota of *Motillmonas* (*P* = 0.018) and *Kurthia* (*P* = 0.003) were significantly higher in shrimp fed high-fat than in shrimp fed low-fat diets. Notable variations exist between dietary lipid and BA levels, including *Vibrio*, *Photobacterium*, *Motillmonas*, and *Kurthia* *(P < 0.05)*.

**Table 9:** Effects of dietary bile acids (BAs) on the intestinal microbiota composition (%) of *Litopenaeus vannam*ei fed diets with different lipid levels.

Items	Phylum					Genus									
	Proteobacteria	Bacteroidota	Firmicutes	Verrucomicrobiota	Planctomycetota	*Vibrio*	*Tenacibaculum*	*Photobacterium*	*Motilimonas*	*Kurthia*	*Bacillus*	*Shewanella*	*Acinetobacter*	*Ruegeria*	*ZOR000610*
**Treatments**^1^															
LFD	86.95^Aa^	3.89^B^	8.15	0.58^Bb^	0.09^b^	79.87^a^	2.14^B^	0.34	0.23^Bb^	0.23^Ab^	6.75	4.6	0.59	0.36^B^	0.098
LF400	89.16^Ba^	8.47^A^	0.818	0.98Ab	0.31^b^	83.08^a^	6.28^A^	2.11	0.33^Ab^	0.04^Bb^	0.21	0.67	0.62	1.75^A^	0.31
HFD	88.06^Ab^	2.30^B^	8.91	0.33^Ba^	0.14^a^	69.06^b^	1.93^B^	12.3	0.04^Ba^	7.69^Aa^	0.72	0.56	5.00	0.50^B^	0.10
HF400	73.92^Bb^	13.37^A^	5.37	5.67^Aa^	0.50^a^	53.97^b^	5.38^A^	0.13	8.08	0.00^Ba^	0.06	0.62	0.09	3.34^A^	3.37
SEM	0.003	0.002	0.184	0.000	0.171	0.001	0.057	0.024	0.003	0.000	0.092	0.198	0.044	0.002	0.174
***P*-value**^2^															
BA	0.034	<0.001	0.063	<0.001	0.492	0.157	0.009	0.080	0.012	0.002	0.091	0.215	0.064	0.001	0.154
Lipid level	0.015	0.323	0.337	<0.001	<0.001	<0.001	0.655	0.093	0.018	0.003	0.14	0.192	0.13	0.091	0.205
BA × Lipid	0.007	0.067	0.489	<0.001	0.498	0.039	0.784	0.025	0.014	0.003	0.159	0.23	0.062	0.147	0.207

SEM = standard error of the mean.

1 LFD, and LF400 represent 7% lipid with 0 and 400 mg/kg BA, respectively; HFD, and HF400, represent 11% lipid with 0 and 400 mg/kg BA, respectively.

2 Statistical significance was set at *P* < 0.05 (*n* = 4). Significant differences between dietary BA levels are indicated by different superscript capital letters within columns (*P* < 0.05), while different superscript lowercase letters denote significant differences between dietary lipid levels (*P* < 0.05), BA × Lipid indicates the BA level and lipid interaction level (*P* < 0.05). The *P* values of linear and quadratic were the differences among groups.

The abundance of microbial taxa among the four groups was assessed using the LDA (score > 4) and the LEfse package. As shown in Fig. 6A and B, this analysis showed significant group differences at the phylum level between Bacteroidota and Verrucomicrobiota in the HF400 group, Proteobacteria in the LF400 group, and Firmicutes in the HFD group. At the genus level, the LFD group contained *Bacillus*; the LF400 group contained *Vibrio*; the HFD group contained *Photobacterium*, *Kurthia*, and *Acinetobacter*; and the HF400 group contained *Akkermansia*, *Motilimonas*, *Ruegeria*, and *ZOR0006*.

#### 3.8.3 Functional prediction and correlation analysis of the intestinal microbiota

The PICRUST2 predicts the functional characteristics of intestinal microbiota in LFD, LF400, HFD, and HF400 groups (Fig. 7A). The LFD400 group exhibited significantly elevated relative abundances in > 90% of predicted pathways, including core metabolisms (carbohydrate, amino acid, and lipid) and specialized pathways (xenobiotic biodegradation and secondary metabolites). In contrast, the LFD and HFD groups showed uniformly low pathway activities without statistical significance (*P* > 0.05), except for a pronounced decrease in secondary metabolite biosynthesis in the HFD group. Strikingly, the HF400 group demonstrated broad downregulation of all pathways compared to the LFD400 group, suggesting a diet-dependent suppression of microbial functionality.

As shown in Fig. 7B, *srebp* gene expression exhibited positive correlations with the relative abundance of *Bacillus* and *Shewanella*, while the relative abundance of *Vibrio* showed negative correlations with both *fabp* and *fas*. Notably, the relative abundance of *Mobilimonas* was only negatively correlated with *fabp* *gene expression*, whereas *Ruegeria* demonstrated positive correlations with both *fabp* and *fas* gene expression . In terms of morphological parameters, the relative abundance of *Tenacibaculum* showed a positive correlation with fold width, the relative abundance of *Vibrio* correlated positively with fold height, and *Kurthia* exhibited negative correlations with both fold width and fold height. Intestinal basal thickness was positively correlated with the relative abundance of *ZOR0006* and *Mobilimonas*, while muscle thickness displayed a positive correlation with *Vibrio* but a negative correlation with the relative abundance of *Mobilimonas*. Interestingly, both microvillus height and microvilli showed negative correlations with the relative abundance of *Photobacterium*, *Kurthia,* and *Acinetobacter*, with microvilli being uniquely positively correlated with *Ruegeria*.

## 4 Discussion

### 4.1 The addition of BA improved shrimp growth performance by regulating lipid metabolism

Recent studies on *L. vannamei* have demonstrated that BA supplementation exerts dose-dependent effects on physiological functions. For instance, BA at 150 to 900 mg/kg improves growth performance and enterohepatic circulation in diets replacing 60% fishmeal (Li et al., 2023a), whereas lower doses (75-350 mg/kg) enhance nutrient utilization efficiency (Wang et al., 2023). Conversely, higher doses (400-800 mg/kg) specifically optimize lipid metabolism and hepatopancreatic health (Li et al., 2022). To systematically evaluate these dose-responsive effects, BA concentrations of 200, 400, and 800 mg/kg were selected to assess impacts on growth, metabolic efficiency, and organ function.

Furthermore, to investigate the interaction between dietary lipid levels and BA efficacy, experimental diets were formulated to contain 7% (low-fat) and 11% (high-fat) CL. Shrimp fed diets supplemented with 400 mg/kg BA exhibited significantly enhanced growth performance compared to those receiving unsupplemented diets or diets supplemented with 200 mg/kg BA. The addition of BA to the feed promotes growth, and similar results were seen in juvenile Pacific white shrimp and common carp (*Cyprinus carpio*) (Li et al., 2023b; Su et al., 2021; Xian et al., 2021). Notably, the growth-promoting effects of BA at 400 mg/kg coincided with improved lipid metabolism, indicating a potential link between BA's growth enhancement and its ability to modulate energy partitioning. In addition, the FCR of shrimp-fed high-fat diets was higher than that of the low-fat group, and consumed too much feed and failed to achieve the corresponding growth effects. There was no notable difference in shrimp growth when fed a high-fat diet, indicating that the *L. vannamei* can tolerate 11% lipid. This is similar to the findings of previous studies (Boujard et al., 2004; Martins et al., 2007), which showed that feeding high-fat diets did not significantly improve shrimp growth. Some previous studies have shown that fish and shrimp-fed high-fat diets show no significant change in growth (Ding et al., 2020a; Zhang et al., 2022a). The high energy in the diet likely led to a reduction in feed consumption and lower intake of other nutrients, which may also contribute to the higher FCR of shrimp in the high-fat group (Wang et al., 2005). The results of shrimp body composition showed that the body lipid content of shrimp fed 400 mg/kg BA was significantly lower compared to shrimp fed 200 mg/kg BA, and the high-fat diet increased the whole body CL content of shrimp. The inclusion of 400 mg/kg of BA significantly enhances shrimp growth and reduces the body lipid content of shrimp. This dual effect—simultaneous growth promotion and lipid reduction—highlights BA's unique role in redirecting metabolic energy from storage to growth processes.

At the molecular level, this effect is mediated through BA's coordinated regulation of key lipid metabolism genes. Specifically, FAS, which encodes genes for rate-limiting enzymes in fatty acid synthesis, is a specific downstream target of SREBP1 (Xu et al., 2019). As a metabolic regulator, AMPK phosphorylates SREBP1, inhibits the movement of these transcription factors into the nucleus (Jung et al., 2011), and ACC serves as another key downstream effector in fatty acid synthesis regulation (Wang et al., 2024). The FABPs constitute a highly conserved family of intracellular lipid chaperones that facilitate fatty acid uptake, transport, and compartmentalization, with particular importance in intracellular lipid trafficking and metabolic regulation (Rawat et al., 2024). Furthermore, SREBPs serve as master transcriptional regulators of lipid homeostasis, primarily controlling the expression of genes involved in cholesterol, fatty acid, and triglyceride biosynthesis (Jung et al., 2011). In the present study, BA supplementation demonstrated tissue-specific regulatory effects on these genes. Although high-fat diets suppressed the gene expression of *fas* in the hepatopancreas, dietary supplementation with 400 mg/kg BA stimulated the gene expression of *fas* and *fabp* in shrimp intestine, while simultaneously reducing the gene expression of *srebp*.

The tissue-specific regulation was particularly evident in the hepatopancreas, where high-fat diets increased the gene expression of *acc*, a pattern resembling obese mice models that show elevated ACC phosphorylation with reduced gene expression of *fas*, promoting fat accumulation (Lee et al., 2023). Interestingly, BA supplementation specifically enhanced the gene expression of *ampk* without affecting the gene expression of *acc* in the hepatopancreas, suggesting a preferential activation of energy-sensing pathways. These findings are supported by previous studies demonstrating that BA supplementation in shrimp feed upregulates the gene expression of *fabp* and *fas* and activates the PPAR-related pathway, thereby promoting lipid catabolism and accelerating lipid digestion (Li et al., 2023a). Consistent with the results, the addition of BA to yellow catfish (*Pelteobagrus fulvidraco*) diets attenuated the gene expression of *srebp* induced by a high-fat diet in the intestine (Li et al., 2024). Taken together, the coordinated upregulation of the gene expression of *fas* and *fabp* coupled with suppression of the gene expression of *srebp* suggests BA promotes a metabolic shift towards enhanced fatty acid utilization rather than storage.

Regarding lipid deposition patterns, excessive dietary lipid was found to promote lipid deposition in shrimp, manifested by an increased number of lipid droplets in the hepatopancreas and elevated body fat content. Similar effects of high-fat diets on lipid accumulation have been reported in other aquatic species, including Nile tilapia (*Oreochromis niloticus*) (Luo et al., 2020), rice field eel (*Monopterus albus*) (Shi et al., 2022), and grass carp (*Ctenopharyngodon idella*) (Tang et al., 2019a). BA supplementation in high-fat diets enhanced shrimp growth and mitigated the growth-inhibitory effects induced by high lipid intake. The feed with an 11% CL; level did not adversely affect the growth performance of the shrimp, but it reduced the shrimp's ability to utilize the feed. In conclusion, this inverse relationship between growth performance and body lipid content underscores BA's role in partitioning dietary energy towards growth rather than storage, although the intrinsic mechanisms through which BA improves fat utilization and alleviates lipid deposition require further elucidation.

### 4.2 The addition of BA reduced lipid deposition in shrimp

The liver serves as a central regulator of lipid metabolism; TG accumulation arises when TG synthesis outpaces export capacity via membrane-associated transport mechanisms (Zhang et al., 2019). Lipoproteins, which transport lipids in the bloodstream, are primarily classified into two major classes: LDL and HDL (Darabi and Kontush, 2022). In this study, supplementation of a high-fat diet with 400 or 800 mg/kg of BA led to a decrease in TG levels in hemolymph, and the addition of BA led to an increase in HDL-C and LDL-C levels. Comparable findings were noted in both grass carp and tilapia (Jiang et al., 2018; Zhou et al., 2018) which suggested that BA supplementation enhances lipoprotein activity in the hemolymph, increases the levels of HDL and LDL, and consequently lowers triglyceride levels in the hemolymph (Sirvent et al., 2004). Moreover, shrimp that were given a high-fat diet exhibited elevated T-CHO levels, and similarly, T-CHO levels in the hemolymph increased following BA supplementation. This is inconsistent with previous findings (Bhusare et al., 2023). The difference lies in the decrease in hemolymph T-CHO levels after BA supplementation. Differences between studies may be caused by different variables, such as the dose of BA (Ding et al., 2020b; Romano et al., 2020), and the protein content of the base formula, etc (Liao et al., 2020; Yu et al., 2019). The current research revealed that shrimp on high-fat diets showed elevated levels of TG, T-CHO, and NEFA in their hepatopancreas relative to those on a low-fat diet. And supplementation of 800 mg/kg BA was effective in reducing TG and T-CHO content in shrimp hepatopancreas. This is consistent with the findings on common carp (*C*. *carpio* L.) and hybrid grouper (Xu et al., 2022a; Yang et al., 2023a). Supplementation with BA notably decreased TG deposition in the hepatopancreas, indicating that adding BA to the diet effectively lowered TG deposition in the hepatopancreas and enhanced LDL activity in the hepatopancreas. Interestingly, this observation was confirmed by subsequent oil-red O staining, which revealed significantly more abundant hepatopancreatic lipid droplets in shrimp fed a high-fat diet compared to those on a low-fat diet. However, the addition of BA to the diet at up to 400 mg/kg led to a significant reduction in lipid droplets in the hepatopancreas similar to what were observed by Tang et al. (2019b) and Hu et al. (2024). The ability of BA to reduce lipid deposition in shrimp may be due to the ability of BA to act as a natural emulsifier, to emulsify fats into particles and convert them into water–soluble complexes, thereby initiating fatty acid uptake (Kiriyama and Nochi, 2019; Wahlström et al., 2016).

Additionally, histological sections serve as an indicator for assessing the health status of the hepatopancreas. In the hepatopancreas, B-cells are secretory cells responsible for toxin excretion, while R-cells are reabsorptive cells involved in digestion and nutrient storage (Hu and Leung, 2007). In the low-fat diet group, the hepatopancreatic structure exhibited disorganized hepatic tubules and swollen B-cells, likely due to vacuolization of the tubule epithelial cells caused by lipid deficiency in the low-fat diet. This finding contrasts with previous studies reporting that high-fat diets reduce R-cell populations, induce B-cell swelling, and decrease antioxidant enzyme activity (Sun et al., 2022). Although the effects of BA were not pronounced in the low-fat group, BA supplementation in high-fat diets improved hepatopancreatic morphology. Specifically, BA increased R-cell abundance and restored the orderly arrangement of hepatic tubules, consistent with previous findings (Yin et al., 2021; Zhang et al., 2022b). These results suggest that BA plays a critical role in maintaining the normal structure of hepatopancreatic cells.

### 4.3 The addition of BA ameliorated ferroptosis in shrimp fed a high-fat diet

High-fat diets cause lipid peroxidation, and direct damage to membrane integrity and cellular components has been demonstrated (Park et al., 2016; Tan and Norhaizan, 2019). Accumulation of lipid peroxides and hyperactivity of free radicals leads to ferroptosis (Gao et al., 2023). This process is exacerbated by the hepatopancreatic lipid deposition observed in this study, creating a direct link between ectopic lipid accumulation and oxidative stress. Therefore, it is necessary to investigate whether the addition of BA to a high-fat diet can alleviate the ferroptosis caused by lipid peroxidation in the shrimp organism. The MDA is a key byproduct of lipid peroxidation, and its level in the animal body can serve as an indirect indicator of lipid peroxidation damage (Fan et al., 2023). Increasing research indicates that high-fat diets significantly elevate the MDA content levels in aquatic animals. For example, the MDA content levels in the plasma and liver of largemouth bass have risen, and similar findings have been observed in large yellow croaker and blunt snout bream (Ding et al., 2020a; Lu et al., 2014; Yin et al., 2021). Interestingly, these findings align with the experimental results. They suggest that the antioxidant system of shrimp is compromised by a high-fat diet, and the addition of BAs can effectively mitigate such damage. This protective effect is consistent with the observed reduction in hepatopancreatic TG content and restoration of HDL/LDL balance, demonstrating BA's systemic antioxidant capacity.

Ferroptosis exhibits mitochondrial ultrastructural alterations, including reduced volume, elevated density, and cristae loss (Xie et al., 2016). This is similar to the results of the present experiment, in which TEM results showed that mitochondrial cristae were disorganized, disappeared, and increased membrane density in the HFD group, whereas mitochondrial morphology and a portion of the mitochondrial cristae could be seen in the group with added BA. Ferritin is the primary iron storage protein in cells and is crucial for maintaining iron homeostasis (Zhang et al., 2021). A high-fat diet was found to cause systemic iron deficiency in mice. Chronic inflammation further reduces iron absorption in the duodenum, while ferritin synthesis requires increased levels of free Fe^2+^ (Sonnweber et al., 2012). This is similar to the finding that shrimp in the HFD group had significantly lower levels of *fer* mRNA expression than the other bile acid-added groups. In addition, *hif* are known to control iron regulatory proteins (Peyssonnaux et al., 2008). Studies have shown that lipid accumulation in *P*. *fulvidraco* is linked to oxidative stress and the induction of *hif*-*1α* (Wang et al., 2021). A previous study found that a high-fat diet activated the hypoxia signaling pathway in adipose tissue of grass carp (Wei et al., 2024). The results of the present study revealed that the hepatic addition of BA was able to significantly reduce the level of *hif-1α* mRNA expression. The ACSL4 is a pivotal enzyme in iron-associated lipid metabolism that promotes ferroptosis by catalyzing the esterification of polyunsaturated fatty acids (PUFAs) into their CoA derivatives, specifically arachidonoyl-CoA (AA-CoA) and adrenoyl-CoA (AdA-CoA) (Kagan et al., 2017). These CoA-esterified PUFAs serve as essential precursors for phospholipid hydroperoxide (PL-OOH) biosynthesis (Mishima and Conrad, 2022). Previous studies have shown that the mRNA expression of *acsl4* increases in largemouth bass under high-fat diets and in silver carp under hypoxic stress-induced liver injury (He et al., 2024; (Zhang et al., 2025). Similarly, in this experiment, the expression of *acsl4* was significantly higher in the high-fat group of shrimp compared to the low-fat group. However, the addition of BA reduced hepatic lipid deposition and alleviated liver damage in the shrimp. These findings suggest that BA may mitigate ferroptosis in hepatopancreatic cells by decreasing lipid accumulation.

Glutathione peroxidase 4 is known to prevent lipid peroxidation, oxygen-specific epitope accumulation, and cell death (i.e., ferroptosis) (Schwärzler et al., 2022). Moreover, GPX4 has been suggested to be a major controller of the lipid peroxidation reaction, using glutathione as a cofactor to reduce lipid peroxidants to lipids, thereby scavenging (Fe^2+^)-dependent ROS, and thus activation of GPX4 prevents ferroptosis (Ursini and Maiorino, 2020). The accumulation of lipid peroxides can activate iron dysregulation as a result of suppressed gene expression of *gpx4* (Chen et al., 2025). Studies have shown that adding a natural antioxidant, sulforaphane, to a high-fat diet can upregulate the expression of the *gpx4* gene in mice (Zhang et al., 2022c). This finding aligns with the restored metabolic parameters. Nevertheless, the gene expression of *gpx4* was uniquely upregulated in response to the high-fat diet. The results of immunofluorescence analysis in this study showed that the relative rate of GPX4 immunofluorescence was significantly higher in the group supplemented with 400 mg/kg BA than in the group supplemented with 0 mg/kg BA, and was higher in the low-fat group than in the high-fat group. Currently, there are experiments showing that mediating GPX4 and thus attenuating ferritin deposition, for example, ouabain can activate GPX4 to alleviate diabetic nephropathy (Zhang et al., 2023) and alleviation of lipid peroxidation-induced non-alcoholic steatohepatitis in mice by increasing hepatic GPX4 with sodium selenite (GPX4 activator) (Qi et al., 2020). This suggests that the addition of BA to high-fat diets is also effective in activating GPX4 protein expression and thus mitigating lipid peroxidation-induced damage.

### 4.4 The addition of BA improved the intestinal barrier function of shrimp-fed high-fat diets

It is well established that the onset of metabolic disorders, including those caused by high-fat diets, often begins with damage to the intestinal barrier (Dai et al., 2022). Key early events in this process involve the disruption of intestinal tight junctions, increased permeability, and metabolic dysregulation of the intestinal microbiota (Vancamelbeke and Vermeire, 2017). In addition, intestinal morphology reflects intestinal health and is used to assess its digestive and absorptive capacity, in which microvilli height and microvilli are important for nutrient absorption (Han et al., 2015; Ye et al., 2019). Previous research indicates that high-fat diets cause blunt snout bream (*Megalobrama amblycephala*), juvenile grass carp (*C*. *idella*), and impairment of intestinal barrier function (Liu et al., 2022; Yu et al., 2020). Similar to the observed high-fat diet-induced hepatopancreatic lipid accumulation, these intestinal changes may constitute another manifestation of systemic metabolic dysregulation. The TEM results from the high-fat group corroborate these observations. Specifically, the epithelial microvilli in the midgut of shrimp fed the high-fat diet were shed and dispersed. The tight junctions were less distinct, intestinal villi height was significantly reduced, and the internal structure of the mitochondria was lost. Furthermore, the histomorphometric sections indicated a significant reduction in fold height, fold width, and muscle thickness in shrimp from the high-fat group. This decrease suggests that high-fat diets induce damage to the intestinal histomorphometry of shrimp. However, there is growing evidence indicating that appropriate BA supplementation effectively protects the intestinal tract (Li et al., 2021). This aligns with the present study's findings, where BA supplementation promoted growth in fold width, fold height, muscle thickness, and microvilli, and mitigated the damage to intestinal morphology caused by high-fat diets. One reason for this is that BA promotes the regeneration of intestinal epithelial cells. They facilitate mucus secretion and the release of nutrient-inducing hormones, which aid in the recovery of damaged epithelial cells and the restoration of intestinal function (Mroz et al., 2018; Sorrentino et al., 2020).

### 4.5 The addition of BA improved the intestinal microbiota composition of shrimp-fed high-fat diets

Intestinal microbial communities are crucial for the proper growth and upkeep of the intestinal barrier's functionality (Alam and Neish, 2018). Chronic high-fat diets induce intestinal dysbiosis in terrestrial and aquatic vertebrates, facilitating opportunistic pathogen colonization, as evidenced by experimental studies (Yoo et al., 2021). This dysbiosis may exacerbate intestinal barrier damage—as observed via TEM and histomorphometric analyses (fold height/width reductions)—potentially creating a vicious cycle of impaired nutrient absorption and metabolic dysfunction. Furthermore, the interaction between BA and intestinal microbiota is complex. Various intestinal microbiota compositions metabolize different BA, and these BA play a crucial role in shaping the intestinal microbiota, particularly under high-fat diet conditions (Yokota et al., 2012). The findings indicated that shrimp fed a high-fat dietexhibited the lowest intestinal microbiota diversity, whereas the HF400 group showed the highest diversity. In addition, intestinal microbial α-diversity indices showed that as the amount of BA added to the diet increased, the diversity indices of Sobs, Shannon, and Simpson were significantly higher in shrimp-fed diets with 400 mg/kg BA than in shrimp-fed diets supplemented with 0 mg/kg BA. The study revealed that the diversity of intestinal microbiota in the group supplemented with 400 mg/kg BA was significantly higher than that of shrimps fed diets supplemented with 0 mg/kg BA. The PCoA analysis further revealed that the curve for the 400 mg/kg BA-supplemented feed group differed from that of the 0 mg/kg BA-supplemented group. This indicates that BA influences the intestinal microflora structure in shrimp. Similar to other studies, the dominant intestinal microbiota in shrimp was composed of five phyla: Proteobacteria, Bacteroidota, Firmicutes, Verrucomicrobia, and Planctomycetota (Li et al., 2023b). It is widely recognized that Bacteroidota and Firmicutes are beneficial in maintaining intestinal health, and many of their genera can efficiently produce short-chain fatty acids (SCFAs), such as *Lactobacillus*, *Lactococcus*, etc (Amandeep Kaur et al., 2019; Zafar and Saier Jr, 2021). An increase in proteobacteria is a potential cause of dysbiosis and up-regulation of pathogenicity (Shin et al., 2015). This study demonstrated that the addition of BA decreased the relative abundance of Proteobacteria and increased Bacteroidota and Verrucomicrobia. These findings are consistent with previous studies (Hu et al., 2024; Su et al., 2021). Interestingly, Bacteroidota enhances the activity of 7α-dehydroxylase and bile salt hydrolases (BSH), which aid in the intestinal conversion of primary BA to secondary BA (Ruiz et al., 2024). Additionally, it is suggested that SCFAs produced by Bacteroidota may stimulate lipolysis, resulting in reduced fat levels around the viscera of fish (Zhou et al., 2018). Verrucomicrobia maintains the integrity of shrimp intestinal epithelial cells, enhances intestinal tight junctions, and promotes the absorption of polysaccharide SCFAs (Tang et al., 2021). The increase in Verrucomicrobia relative abundance promoted by BA addition is consistent with prior reports (Li et al., 2023b). Previous studies have demonstrated that BA exerts a selective bacterial inhibitory effect. Bacteria tolerant to high concentrations of BA often show a relative increase in abundance, while those intolerant to BA may experience changes in their relative abundance (Zhang et al., 2022b). In experiments with grass carp, supplementation with lithocholic acid led to a decrease in the relative abundance of Firmicutes. Similarly, this study observed comparable results (Xiong et al., 2019).

*Vibro*, *Tenacibaculum*, *Photobacterium*, *Motilimona*s, and *Kurthia* are the genera of first-order dominant bacteria. In the realm of potential pathogens, particularly among aquatic organisms, *Vibrio* holds significant importance due to its pervasive pathogenicity in fish and shrimp hosts (Nurliyana Mohamad et al., 2019). This study demonstrated that the high-fat diet group showed a lower *Vibrio* abundance compared to the low-fat group. Notably, *Vibrio* exhibited a negative correlation with lipid metabolism-related genes. As a well-documented pathogenic genus (Xie et al., 2020), *Vibrio* may be inhibited by excessively long-chain fatty acids (LCFAs) in HFD, since most *Vibrio* species lack efficient enzymatic systems for LCFA utilization (Bergsson et al., 2001; Mil-Homens et al., 2016).

Moreover, Dietary BA supplementation significantly altered the intestinal microbiota composition in shrimp, notably increasing the relative abundance of *Tenacibaculum, Motilimonas*, and *Ruegeria* while decreasing *Kurthia* populations. *Tenacibaculum* has been well-documented as a pathogenic genus in shrimp intestinal microbiota (Zhou et al., 2019). While *Motilimonas* has been suggested to associate with hepatopancreatic amino acid metabolism in shrimp (Yu et al., 2022), neither genus has been previously reported to interact with bile acid metabolism. This enrichment may indicate a pathogen-favorable state induced by bile acids, which could in turn heighten disease susceptibility in shrimp. In contrast, *Ruegeria* plays beneficial roles in maintaining intestinal homeostasis and inhibiting marine pathogens (Barreto-Curiel et al., 2018). *Ruegeria* abundance correlated positively with the expression of lipid metabolism genes (*fabp* and *fas*) and microvilli integrity, supporting its previously established role in promoting intestinal health (Zhang et al., 2014). Functional profiling revealed distinct diet-dependent responses to BA supplementation. In shrimp fed the low-fat diet, BA administration significantly enhanced activity in nearly all metabolic pathways, including lipid metabolism, amino acid metabolism, energy metabolism, and biosynthesis of secondary metabolites. This comprehensive upregulation likely stems from synergistic interactions between dietary lipids and BA-modulated microbial metabolic activities, thereby improving nutrient digestion and absorption efficiency in the shrimp intestine (Bao et al., 2024). Notably, compared to the LF400 group, the high-fat diet with BA supplementation (HF400 group) exhibited marked downregulation of the majority of metabolic pathways, with the notable exception of secondary metabolite biosynthesis, which remained relatively stable. This specific pattern suggests that under high-fat dietary conditions, BA's regulatory effects on gut microbiota may preferentially focus on secondary metabolite synthesis as a key metabolic node.

## 5 Conclusion

In conclusion, long-term feeding of high-fat diets does not affect shrimp growth but may result in hepatopancreatic lipid accumulation. Additionally, high-fat diets disrupted lipid metabolism, compromised intestinal health, and altered the intestinal microbiota structure. This study further demonstrated that supplementing high-fat diets with BA reduces overall body lipids in shrimp and effectively mitigates these adverse effects. Bile acid also activates GPX4, which helps counteract lipid peroxidation damage caused by high-fat diets. However, the mechanisms underlying ferroptosis in shrimp require further investigation to enhance our understanding of BA-induced ferroptosis.

## Credit Author Statement

**Kangyuan Qu:** Writing – original draft, Formal analysis, Conceptualization. **Tengfei Zhao:** Resources, Methodology. **Yufei Chen:** Resources, Methodology. **Yunzhen Li:** Resources, Methodology. **Beiping Tan:** Resources, Methodology. **Shiwei Xie:** Writing – review & editing, Supervision, Project administration, Investigation, Funding acquisition.

## Declaration of competing interest

We declare that we have no financial and personal relationships with other people or organizations that can inappropriately influence our work, and there is no professional or other personal interest of any nature or kind in any product, service and/or company that could be construed as influencing the content of this paper.
