# Supplementary material for: Bile acid supplementation in a high-fat diet improved growth performance, lipid deposition, ferroptosis, and intestinal health in Pacific white shrimp (Litopenaeus vannamei)
Source: Anim Nutr. 2026 May 7;26:334–53. doi: 10.1016/j.aninu.2025.09.020 (PMC13293663; doi:10.1016/j.aninu.2025.09.020)
Supplement: Multimedia component 1 [file mmc1.docx]

**Table S1** Premix formulations (mg/kg).

| Premix formulations | Lipid levels, % |  |
| --- | --- | --- |
|  | 7 | 11 |
| Lysine | 2.958 | 2.881 |
| Methionine | 1.296 | 1.245 |
| As | 0.022 | 0.047 |
| Cd | 1.302 | 1.811 |
| Pb | 0.029 | 0.022 |
| Hg | 0.001 | 0.001 |
| Cr | 0.065 | 0.049 |
| F | 0.183 | 14.854 |
| Fe | 260.112 | 216.252 |
| Ma | 42.061 | 37.754 |
| I | 3.431 | 7.681 |
| Co | 1.356 | 1.341 |
| Se | 0.856 | 1.360 |
| Cu | 36.388 | 36.347 |
| Zn | 110.957 | 110.270 |
| Vitamin A | 13.887 | 14.859 |
| Vitamin D_3_ | 780.051 | 763.363 |
| Vitamin E | 182.154 | 190.679 |
| Vitamin K | 28.395 | 27.327 |
| Thiamin-B_1_ | 43.915 | 40.110 |
| Riboflavin-B_2_ | 40.815 | 38.891 |
| Pyridoxine-B_6_ | 52.131 | 50.993 |
| Folic acid-B_9_ | 26.341 | 25.505 |
| Vitamin B_12_ | 0.904 | 0.525 |
| Vitamin C | 528.471 | 517.165 |
| Ca | 4.641 | 4.969 |
| P | 2.953 | 3.200 |
| Ca/P (index) | 1.571 | 1.553 |

**Table S2** Primers used for quantitative real-time PCR.

| Genes | Forward primers (5’-3’) | Reverse primers (3’-5’) | Accession No. |
| --- | --- | --- | --- |
| *ef-1α* | GTATTGGAACAGTGCCCGTG | ACCAGGGACAGCCTCAGTAAG | JF288785.1 |
| *fer* | GAGTACCTTCAAGCGAACC | CAGAAGCCAACACCAGAT | Zhou et al., 2008 |
| *hoi* | GCATGGCAGTGACCGAGATTGA | GTCGCTGCTTCGTCTCCTCATC | LOC113823598 |
| *hif-1a* | GCAATCAGCTTTCTGCTCCC | GGTCAGGGTCCATCAATAGC | LOC113823783 |
| *acc* | CGGCAGACAACATCCATACCACAG | GCAACCAGCGAGAGCAGTAACC | XM027360190 |
| *ampk* | TCAGAGGAGGAGCAGGAAC | CCCGAGGTCTAATAGGCAC | KP272117.1 |
| *fas* | AGGTGGTTATGCTGCCGTCAA | TCTCCTCTTCGCCGCTGGTA | HM595630.1 |
| *fabp* | CGGCGATACCTACACAA | AGCATCTTGTCGTCGGT | DQ398572.1 |
| *srebp* | AGGCGAGAAACGCAACT | GAGGGTGATGGAGGCAG | MG770374.1 |
| *acsl4* | TGTTCGGCTGCGTAATCCAAG | CGAGGAAATGGCTTGTCCGT | LOC113810703 |

**Table S3** Abbreviations and full names of genes.

| Abbreviations | Full names |
| --- | --- |
| *acc* | Acetyl-CoA carboxylase |
| *acsl4* | Acyl-CoA synthetase long-chain family member 4 |
| *ampk* | AMP-activated protein kinase |
| *ef-1α* | Elongation factor 1-alpha |
| *fabp* | Fatty acid-binding protein |
| *fas* | Fatty acid synthase |
| *fer* | Ferritin |
| *gpx4* | Glutathione peroxidase 4 |
| *hif-1a* | Hypoxia-inducible factor 1-alpha |
| *hoi* | Hemocyanin oxygen-insensitive subunit |
| *srebp* | Sterol regulatory element-binding protein |

**Table S4** Data pre-processing statistics and quality control of 16S rDNA gene sequencing.

| Samples | Raw PE^1^ | Clean PE^2^ | Raw tags^3^ | Clean tags^4^ | Chimera^5^ | Effective tags^6^ | Effective ratio^7^, % |
| --- | --- | --- | --- | --- | --- | --- | --- |
| LF1-1 | 123,090 | 123,043 | 122,537 | 122,406 | 17,534 | 104,872 | 85.20 |
| LF1-2 | 76,466 | 76,428 | 76,055 | 75,969 | 8981 | 66,988 | 87.60 |
| LF1-3 | 124,211 | 124,147 | 123,566 | 123,391 | 19,048 | 104,343 | 84.00 |
| LF1-4 | 75,536 | 75,503 | 75,164 | 75,095 | 1302 | 73,793 | 97.69 |
| LF1-5 | 126,227 | 126,169 | 125,722 | 125,629 | 7575 | 118,054 | 93.53 |
| LF1-6 | 112,206 | 112,153 | 111,786 | 111,648 | 5374 | 106,274 | 94.71 |
| LF1-7 | 90,126 | 90,076 | 89,116 | 88,997 | 8126 | 80,871 | 89.73 |
| LF1-8 | 130,272 | 130,213 | 129,682 | 129,530 | 13,418 | 116,112 | 89.13 |
| LF3-1 | 64,531 | 64,493 | 64,132 | 64,025 | 1405 | 62,620 | 97.04 |
| LF3-2 | 87,406 | 87,359 | 86,621 | 86,509 | 7119 | 79,390 | 90.83 |
| LF3-3 | 121,154 | 121,100 | 120,543 | 120,389 | 9995 | 110,394 | 91.12 |
| LF3-4 | 137,566 | 137,492 | 136,797 | 136,603 | 11,826 | 124,777 | 90.70 |
| LF3-5 | 118,814 | 118,754 | 118,182 | 118,037 | 7221 | 110,816 | 93.27 |
| LF3-6 | 120,510 | 120,449 | 119,831 | 119,590 | 6144 | 113,446 | 94.14 |
| LF3-7 | 103,331 | 103,272 | 102,799 | 102,173 | 3143 | 99,030 | 95.84 |
| LF3-8 | 114,930 | 114,878 | 113,974 | 113,839 | 7357 | 106,482 | 92.65 |
| HF5-1 | 128,707 | 128,662 | 128,063 | 127,947 | 10,698 | 117,249 | 91.10 |
| HF5-2 | 111,870 | 111,779 | 108,927 | 108,786 | 8384 | 100,402 | 89.75 |
| HF5-3 | 90,288 | 90,249 | 89,426 | 89,325 | 3419 | 85,906 | 95.15 |
| HF5-4 | 133,102 | 133,060 | 132,435 | 132,084 | 7015 | 125,069 | 93.96 |
| HF5-5 | 102,687 | 102,638 | 102,032 | 101,929 | 9566 | 92,363 | 89.95 |
| HF5-6 | 136,219 | 136,166 | 135,757 | 135,480 | 5120 | 130,360 | 95.70 |
| HF5-7 | 57,763 | 57,748 | 57,499 | 57,449 | 4035 | 53,414 | 92.47 |
| HF5-8 | 118,276 | 118,217 | 117,639 | 117,447 | 11,085 | 106,362 | 89.93 |
| HF7-1 | 124,588 | 124,520 | 123,896 | 123,440 | 9255 | 114,185 | 91.65 |
| HF7-2 | 119,377 | 119,316 | 118,801 | 118,389 | 8943 | 109,446 | 91.68 |
| HF7-3 | 125,278 | 125,205 | 124,612 | 124,184 | 9218 | 114,966 | 91.77 |
| HF7-4 | 130,273 | 130,211 | 129,661 | 129,318 | 15,735 | 113,583 | 87.19 |
| HF7-5 | 127,337 | 127,279 | 126,710 | 126,311 | 15,521 | 110,790 | 87.01 |
| HF7-6 | 81,192 | 81,158 | 80,767 | 80,487 | 8184 | 72,303 | 89.05 |
| HF7-7 | 98,069 | 98,022 | 97,562 | 97,118 | 6878 | 90,240 | 92.02 |
| HF7-8 | 127,629 | 127,582 | 126,925 | 126,477 | 14,670 | 111,807 | 87.60 |
| Average | 110,595 | 110,542 | 109,913 | 109,688 | 8853 | 100,835 | 91.35 |

PE = pair-end.

^1^Raw PE, logarithm of raw pair-end reads.

^2^Clean PE, number of pairs of high-quality pair-end reads obtained after quality control filtering.

^3^Raw tags, number of raw tags assembled by overlap.

^4^Clean tags, the number of high qualityTags obtained after tags quality control.

^5^Chimera, the number of chimeric tags detected during operational taxonomic units (OTU) clustering.

^6^Effective tags, the number of high-quality tags after removing chimeras, i.e., effective tags for subsequent analyses.

^7^Effective ratio (%), the number of high quality tags as a percentage of the original PE reads.

**Tab****le S5** Number and abundance of Tags in 16S rDNA gene sequencing.

| Samples | Tags number | Total length, bp | Max length, bp | Average length, bp | Min length, bp | N50, bp | N90, bp |
| --- | --- | --- | --- | --- | --- | --- | --- |
| LF1-1 | 104,872 | 48,468,533 | 469 | 675 | 206 | 466 | 461 |
| LF1-2 | 66,988 | 31,130,354 | 478 | 715 | 237 | 466 | 465 |
| LF1-3 | 104,343 | 48,308,204 | 469 | 693 | 224 | 466 | 461 |
| LF1-4 | 73,793 | 34,341,139 | 469 | 706 | 237 | 466 | 466 |
| LF1-5 | 118054 | 54,814,121 | 476 | 713 | 237 | 466 | 461 |
| LF1-6 | 106,274 | 49,388,673 | 478 | 705 | 227 | 466 | 466 |
| LF1-7 | 80,871 | 37,557,385 | 473 | 705 | 232 | 466 | 461 |
| LF1-8 | 116,112 | 53,872,765 | 470 | 698 | 228 | 466 | 461 |
| LF3-1 | 62,620 | 29,120,078 | 468 | 671 | 203 | 466 | 466 |
| LF3-2 | 79,390 | 367,666,38 | 469 | 700 | 231 | 466 | 461 |
| LF3-3 | 110,394 | 51,278,443 | 470 | 707 | 237 | 466 | 461 |
| LF3-4 | 124,777 | 57,829,022 | 469 | 678 | 209 | 466 | 461 |
| LF3-5 | 110,816 | 51,529,495 | 474 | 677 | 203 | 466 | 465 |
| LF3-6 | 113,446 | 52,686,035 | 473 | 710 | 237 | 466 | 461 |
| LF3-7 | 99,030 | 46,071,428 | 469 | 697 | 228 | 466 | 466 |
| LF3-8 | 106,482 | 49,426,858 | 469 | 701 | 232 | 466 | 461 |
| HF5-1 | 117249 | 54,499,741 | 469 | 697 | 228 | 466 | 461 |
| HF5-2 | 100,402 | 46,611,702 | 478 | 703 | 225 | 466 | 466 |
| HF5-3 | 85,906 | 40,008,810 | 470 | 707 | 237 | 466 | 466 |
| HF5-4 | 125,069 | 56,270,011 | 472 | 692 | 220 | 442 | 441 |
| HF5-5 | 92,363 | 42,983,384 | 477 | 700 | 223 | 466 | 466 |
| HF5-6 | 130,360 | 59,244,365 | 470 | 681 | 211 | 456 | 441 |
| HF5-7 | 53,414 | 24,838,476 | 469 | 767 | 298 | 466 | 466 |
| HF5-8 | 106,362 | 49,372,007 | 478 | 700 | 222 | 466 | 461 |
| HF7-1 | 114,185 | 52,686,537 | 472 | 678 | 206 | 465 | 444 |
| HF7-2 | 109,446 | 50,299,593 | 478 | 684 | 206 | 461 | 441 |
| HF7-3 | 114,966 | 52,875,927 | 478 | 695 | 217 | 461 | 441 |
| HF7-4 | 113,583 | 52,250,014 | 478 | 695 | 217 | 461 | 441 |
| HF7-5 | 110,790 | 50,949,996 | 478 | 695 | 217 | 461 | 441 |
| HF7-6 | 72,303 | 33,441,536 | 477 | 688 | 211 | 466 | 460 |
| HF7-7 | 90,240 | 41,605,177 | 478 | 684 | 206 | 466 | 442 |
| HF7-8 | 111,807 | 51,523,163 | 478 | 695 | 217 | 464 | 442 |
| Average | 100,835 | 46,626,550 | 473 | 697 | 224 | 464 | 457 |


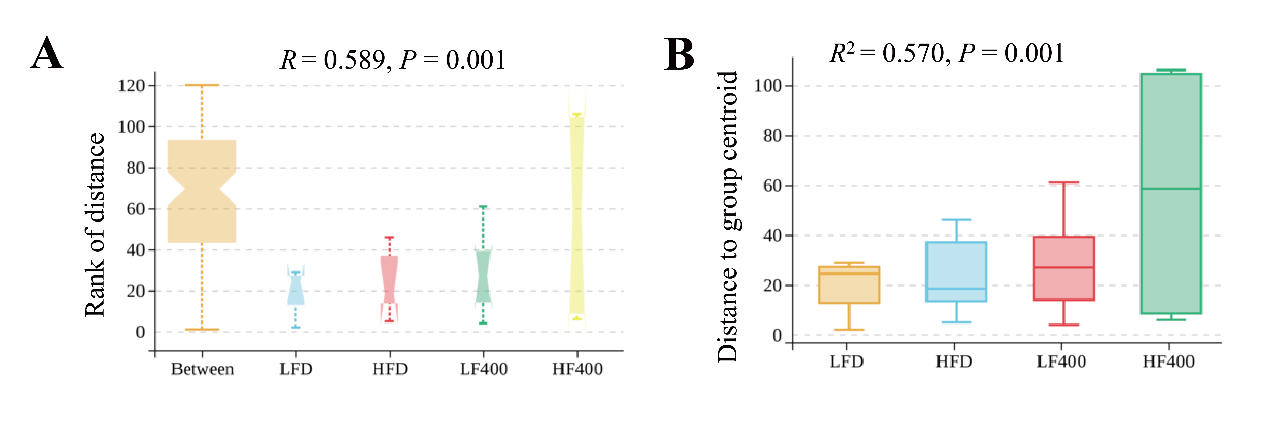


**Fig. S1** Statistical assessment of intestinal microbiota dissimilarity among dietary treatment groups. (A) Analysis of similarities (ANOSIM) result (between-group dissimilarity, *R* = 0.589). (B) PERMANOVA (Adonis) analysis (*R*^2^ = 0.570, *P* = 0.001). ANOSIM and PERMANOVA are multivariate statistical tests used to compare group differences based on dissimilarity distances. LFD and LF400 represent 7% lipid with 0 and 400 mg/kg bile acid (BA), respectively; HFD and HF400 represent 11% lipid with 0 and 400 mg/kg BA, respectively.
